# Stochastic Grey Wolf Optimization for Hyperparameter Tuning of LSTM and RNN Models in Energy Forecasting

**DOI:** 10.1038/s41598-026-56787-w

**Published:** 2026-06-13

**Authors:** Omsaeed Ahmed Albser, Mourad R. Mouhamed, Salma A. Shatta, Nasser H. Sweilem, Ashraf Darwish

**Affiliations:** 1 Department of Mathematics, Faculty of Science, Capital University (Formerly Helwan), Cairo, Egypt; 2 Department of Mathematics, Faculty of Science, Cairo University, Cairo, Egypt

**Keywords:** Photovoltaic power forecasting, Recurrent Neural Networks (RNN), Long Short-Term Memory (LSTM), Hyperparameter optimization, Grey Wolf Optimizer (GWO), Stochastic Grey Wolf Optimizer (SGWO), Time-series forecasting, Energy science and technology, Engineering, Mathematics and computing

## Abstract

Accurate photovoltaic (PV) power forecasts are required to support the stable and efficient incorporation of solar power into modern electric power grids. Even though the use of recurrent neural network models such as RNNs and LSTMs for time series forecasting has proven successful, these model’s ability to make accurate predictions is heavily influenced by proper hyper parameter selection. Therefore, the goal of this research is to introduce a stochastic grey wolf optimizer (SGWO) based hyper-parameter optimization system for RNN and LSTMs used for predicting PV power. A stochastic grey wolf optimizer will be added to the basic grey wolf optimizer to enhance the search capabilities of the algorithm. This new stochastic grey wolf optimizer introduces randomness into the optimization process which can help prevent premature convergence and increase exploration within the problem space. The performance of the proposed SGWO-based system will be tested on a real world PV data set. The comparison will include results from manual tuning, random searching, and standard GWO. Performance metrics will consist of root mean squared error (RMSE), mean absolute error (MAE), and coefficient of determination (R2) and will be used to evaluate how well each of the algorithms performed when making out-of-sample predictions. Results from testing showed that the SGWO-LSTM configuration produced the highest total out-of-sample prediction accuracy; MAE = 0.018, RMSE = 0.041, R^2^ = 0.978.

## Introduction

The need for improved Photovoltaic (PV) power forecasting is largely driven by the rapid growth in adoption of solar energy. Solar energy is an essential component of long term sustainable development and will play a large role in future energy security. However, due to solar’s natural variability and reliance on weather conditions it creates significant uncertainty regarding grid reliability, reserve scheduling and real-time power balancing. Therefore, accurate short and medium term forecasts of PV output are necessary to reduce operational uncertainty, improve dispatch efficiencies, and enable the reliable incorporation of solar output into modern electrical grids^1,2^.

Deep Learning (DL) models have gained prominence in solar forecasting in recent years primarily due to their ability to model complex, nonlinear relationships within time series datasets. Among DL models used for modeling sequential solar irradiance and PV power outputs are Recurrent Neural Network (RNN), Long Short Term Memory (LSTM), etc. These architectures show particular promise at modeling sequential patterns that contain both short and long range temporal autocorrelations^3,4^. Additionally, compared to many other traditional statistical or machine learning based methodologies, RNN/LSTM architectures offer more flexibility in modeling the complex nonlinear behavior of PV production^2,5^.

While RNN and LSTM models show great promise as tools for modeling sequential PV output and solar irradiance, their predictive capabilities remain highly dependent upon optimal selection of various hyperparameters including the learning rate, the size of the hidden layers, the number of layers, and dropout rates. Poor selection of these hyperparameters may result in very slow convergence times, poor generalization abilities, unstable training processes and/or excessive overfitting; all which contribute to reduced levels of forecast accuracy^5,6^. As such, Hyperparameter Optimization (HPO) has become a key step in developing reliable DL based forecasting systems. Traditional methods for performing HPO include manual optimization and random search techniques. Both of these methods suffer from inefficiencies when searching through high dimensional non convex search spaces^7,8^. Meta-heuristics represent alternative HPO strategies that can strike a better balance between the exploratory properties of a search strategy and the exploitative properties of a search strategy in solving complex optimization problems. One meta-heuristic method gaining popularity as a tool for HPO is the Grey Wolf Optimizer (GWO). This method represents one of several swarm based algorithms being proposed as viable alternatives for automating HPO tasks due to its simplicity and competitive search results^9^.

### Research gap, novelty, and contributions

Although recurrent neural network (RNN)-based architectures and metaheuristic optimization methods have each had their own successes with photovoltaic (PV) forecasting, the current literature has relatively little evidence regarding a unified framework that systematically compares RNN and LSTM based architectures in PV forecasting via hyperparameter optimization using a methodological framework that includes deterministic swarm search^5,6,10^. Because it is a model with simple architecture and competitive search behavior, the canonical Grey Wolf Optimizer (GWO) continues to be popular. However, the deterministic nature of its encircling mechanism along with the linear decrease in the control parameter can lead to reduced population diversity and an increased risk of premature convergence in nonlinear or multimodal optimization spaces^9,11,12^. Therefore, motivated by the need to address the aforementioned shortcomings, the current paper introduces stochastic Grey Wolf Optimizer (SGWO), which will be applied for hyperparameter optimization in deep-learning-based PV forecasting. The most important aspect of the proposed framework is the introduction of stochastic perturbation into the standard GWO search process to increase exploration while retaining leader-guided structure, along with evaluating this strategy directly for recurrent PV forecasting under a common experimental protocol. Thus, the main contributions of this study are three-fold: first, development of an SGWO based framework for hyperparameter optimization using both RNN and LSTM models in PV forecasting; second, comparative evaluation of four different strategies for selecting hyperparameters including manual tuning as a baseline with fixed parameters, Random Search implemented through Keras Tuner, canonical GWO, and proposed SGWO; third, empirical assessment of performance of real world PV data forecasting via use of standard regression metrics such as MSE, RMSE, MAE, and R². Unlike several recent versions of improved or stochastic Grey Wolf Optimizer variants that introduce mutation operators, hybrid randomization schemes or modified search rules for general optimization problems^11,12^; the SGWO developed in the current paper preserves the hierarchical nature of the original GWO^9^ but modifies the update mechanism by injecting independent Gaussian perturbations into each of the three components before averaging. This design strengthens exploration, maintains population diversity, reduces risk of premature convergence and retains structural simplicity of original GWO. Additionally, where many improved GWO studies have been validated on benchmark functions or generic optimization tasks only recently^11,12^, the present work validates SGWO within a unified photovoltaic forecasting framework which compares manual tuning, Random Search, canonical GWO and SGWO for both RNN and LSTM architectures under same data split, training budget and evaluation metrics.

## Literature review

The evolution of research on photovoltaic (PV) forecasting has changed from traditional statistical methods toward both data driven and deep learning based models. The move toward using accurate PV forecasting is important for integrating renewable energy into a grid, as inaccuracies in forecasts can impact how efficiently a grid can be balanced, what reserves need to be scheduled, and ultimately how efficient an entire system will operate^2,13^.

Specifically, many researchers have studied different types of recurrent neural network architectures that include RNN’s and LSTM networks. These architectures are able to model non-linear temporal relationships and sequence patterns within time series data sets of solar irradiance and PV output^3,5^ whereas some of the more traditional models used today such as ARIMA and Support Vector Regression have less ability to model the dynamic PV generation process^2,5^.

Hyperparameters in recurrent DLs still heavily depend on their setting to predict the performance of them. The learning rate, size of hidden layer, numbers of layers, drop out ratio, etc., will significantly affect how fast the model converges, how stable it is, and how well the model generalize. Therefore, Hyperparameter Optimization (HPO) is an essential part of developing a DL model. Traditional methods of HPO include Manual Tuning, Grid Search, Random Search which becomes less efficient and/or computationally expensive in high dimensional search spaces^7,8^. Metaheuristics inspired from nature and swarms became popular alternatives since they don’t need gradient information to navigate through complex optimization landscape^5,9^.

The Grey Wolf Optimizer (GWO), among those metaheuristics, attracts researchers’ attentions due to its simple concept, strong search ability and few required parameters to set^9^. Researchers apply GWO into many fields of optimizations like forecasting related tasks and power systems application^11^. However, traditional GWO suffer from loss of population diversity and premature convergence problems caused by its deterministic encircling mechanism and decrease linearly control parameters^9,12^. In order to solve the above mentioned problems, researchers proposed several stochastic and hybrid GWOs to improve the balance between exploration and exploitation. Those studies also show that introducing stochasticity in search mechanisms could improve robustness, reliability of convergence and overall optimization quality in complex ML tasks^11,12^. In addition, some researchers have developed temporal feature engineering techniques (for example cyclical encoding technique with LSTMs based modeling) to better represent the cyclicality of solar generation^14^. Furthermore, the recent study using optimized ML models with hybrid/advanced optimizers to improve performance of models like XGBoost, LightGBM and Extreme Learning Machine demonstrated that the current metaheuristics driven model calibration can be applied to other complex prediction tasks. Though this research was not specifically focused on PV forecasting, the results from the previous studies showed that modern metaheuristics variants can help improve efficiency of ML model tuning when traditional search procedures become less efficient in complicated search space^15–17^. Thus, the previous trends of utilizing metaheuristics variants in the field of ML demonstrate that there is sufficient foundation for researching different stochastic optimizer designs in PV forecasting task.

To provide a structured comparison of the most relevant literature, Table 1 summarizes representative studies in solar PV forecasting and related optimization research, with emphasis on recurrent deep-learning models, hyperparameter optimization, metaheuristic tuning, and methodological limitations.

**Table 1:** Comparative review of representative solar PV forecasting studies and the methodological positioning of the present work.

Study	PV forecasting	RNN/LSTM	Deep learning	Uses HPO	Metaheuristic HPO	GWO-family	Real-world PV data	Main limitation
Jailani et al. (2023)^4^	✓	✓	✓	*Limited*	✘	✘	✓	Recurrent forecasting is effective, but tuning analysis remains limited.
Bacher et al. (2009)^13^	✓	✘	✘	✘	✘	✘	✓	Operational statistical forecasting; no deep learning or HPO.
Inman et al. (2013)^2^	✓	✘	✘	✘	✘	✘	✘	Review paper; highlights heterogeneity but does not offer unified benchmarking.
Rajagukguk et al. (2020)^3^	✓	✓	✓	*Partial*	✘	✘	*Varies*	Reviews deep-learning models but not a reproducible HPO framework.
Ahmed et al. (2020)^5^	✓	*Partial*	✓	*Partial*	✘	✘	*Varies*	Optimization trends are reviewed, but comparative tuning protocols remain weak.
Agga et al. (2022)^6^	✓	✓	✓	✓	✘	✘	✓	Strong short-term PV accuracy, but limited comparison of HPO strategies.
Yu et al. (2024)^10^	✓	✓	✓	*Review-level*	✘	✘	*Varies*	Broad PV review; does not test stochastic swarm-based HPO experimentally.
Mirjalili et al. (2014)^9^	✘	✘	✘	✓	✓	✓	✘	Foundational GWO paper; not specific to PV forecasting or deep learning.
Li et al. (2022)^12^	✘	✘	✘	✓	✓	✓	✘	Improved GWO validated on optimization tasks; PV-specific HPO evidence remains limited.
Qiu et al. (2024)^11^	✘	✘	✘	✓	✓	✓	✘	Recent improved GWO; transfer to PV forecasting must still be demonstrated.
This study	✓	✓	✓	✓	✓	✓	✓	Single-site validation; broader multi-site generalization remains future work.

Where ✓ indicates that feature explicitly present; ✘ indicates that a feature is not present; Partial / Varies / Review-level indicates that a feature is discussed or partially addressed, but not implemented as a unified experimental framework.

The literature has shown that there is a high use of deep learning architectures (especially Recurrent) for PV forecasting. However, there are still three areas where the reviewed literature lacks in providing evidence to support an area of further research. The first is in regards to developing models, optimizing hyperparameters, and doing so as part of a unifying and systematic process. The second limitation is that metaheuristics have been less systematically studied for their performance in solar forecasting compared to other search methods. Lastly, very few authors have made a direct comparison with respect to fixed baseline, tuner based search, standard GWO, and random GWO type variations using the exact same PV site. The gaps identified by this analysis motivated the current study.

## Methodology

**Fig. 1:**
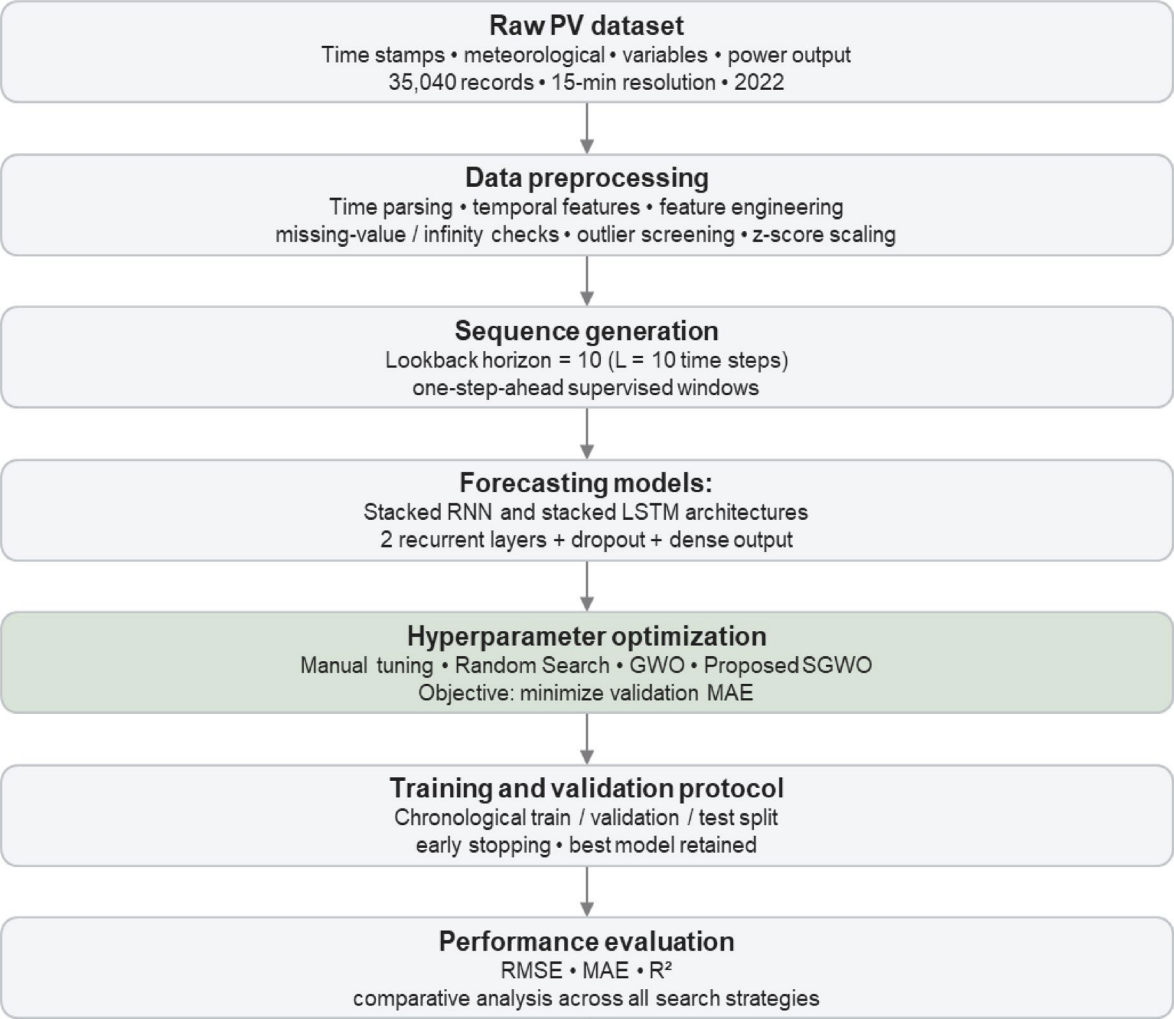
Conceptual workflow of the proposed photovoltaic forecasting framework.

To provide a clear overview of the proposed photovoltaic forecasting framework, the methodological workflow adopted in this study is illustrated in Fig. 1. The framework integrates data preprocessing, sequence generation, recurrent neural network modeling, and hyperparameter optimization using both conventional and metaheuristic search strategies. The overall pipeline is designed to systematically evaluate how different hyperparameter optimization approaches affect the forecasting performance of RNN and LSTM architectures.

Figure 1 provides the overall conceptual workflow of the proposed photovoltaic forecasting framework, while the implementation details of each stage are described in the corresponding methodology subsections.

### Dataset description

The data set we employed in our research has been made available by IEEE Data Port as “Renewable Energy Output Dataset of a Wind Turbine and Photovoltaic Station in Northern China”^18^. Only photovoltaic-related records along with the relevant meteorological factors have been utilized to develop the solar energy prediction model.

Data collection spanned from 1 Jan. 2022 through 31 Dec. 2022 at a sampling frequency of 15 min and therefore, yielded 35,040 pre-sequence generation readings.

Actual Power Generation (p.u.) serves as the target variable; raw predictors are component temperature, air temperature, atmospheric pressure, relative humidity, total radiation, direct radiation, and diffuse radiation. These variables exhibited daily and seasonally correlated patterns that reflect strong temporal dependencies among the data which make them particularly suitable for RNN type models for time series forecasting. Following processing, extraction of temporally correlated features, and development of additional features based on those extracted, the processed design matrix was then utilized to generate all of the forecast sequences for both the RNN-type and LSTM-type experiments (Table 2).

**Table 2:** Raw variables used in the photovoltaic dataset.

Variable	Unit	Role
Time	timestamp	Index / temporal reference
Component temperature	°C	Input
Ambient temperature	°C	Input
Pressure	hPa	Input
Humidity	%	Input
Total radiation	W/m^2^	Input
Direct radiation	W/m^2^	Input
Diffuse radiation	W/m^2^	Input
Actual power generation	p.u.	Target

### Data preprocessing

To create a multivariate forecasting data set with reliability through a full end-to-end pre-processing pipeline, this process has been performed on the raw PV/meteorology data. The pipeline derives temporal and interaction attributes, as well as other derived attributes, to enhance the input representations. Examples include hour, month, day-of-year, quarter, cyclic encoding for variables with periodic characteristics, the temperature-difference variable, a variable indicating efficiency due to irradiance, ratio of direct-to-total irradiance, ratio of diffuse-to-total irradiance, normalized pressure, a squared humidity variable, and a temperature-humidity interaction attribute. It is anticipated that these enhanced inputs will enable the recurrent forecasting models to capture seasonal and intraday nonlinear relationships that would have otherwise remained unaccounted for by relying solely on the raw input variables^10,14^.

#### Temporal feature engineering and exploratory analysis

Given the strong diurnal and seasonal periodicity of solar PV generation, the timestamp variable was first converted into a datetime format and decomposed into calendar-based descriptors, including hour, day, month, day of year, and quarter. To preserve the cyclical nature of periodic temporal effects, the hour, month, and day-of-year components were encoded using sine and cosine transformations. This representation allows the forecasting models to treat temporally adjacent values, such as 23:00 and 00:00 or December and January, as neighboring points in the feature space rather than distant numerical values, consistent with recent PV forecasting studies such as^14^.

1 $${Hour}_{sin}=\mathrm{sin}\left(\frac{2\pi \times Hour}{24}\right)$$

2 $${Hour}_{cos}=\mathrm{cos}\left(\frac{2\pi \times Hour}{24}\right)$$

3 $${Month}_{sin}=\mathrm{sin}\left(\frac{2\pi \times Month}{12}\right)$$

4 $${Month}_{cos}=\mathrm{cos}\left(\frac{2\pi \times Month}{12}\right)$$

5 $${DayOfYear}_{sin}=\mathrm{sin}\left(\frac{2\pi \times Day Of Year}{365}\right)$$

6 $${DayOfYear}_{cos}=cos(\frac{2\pi \times DayOfYear}{365})$$

In addition to the temporal descriptors, several physically meaningful engineered variables were constructed to better represent photovoltaic conversion behavior. These include the temperature difference between component and ambient conditions, a radiation-efficiency indicator, the direct-to-total and diffuse-to-total radiation ratios, normalized atmospheric pressure, a squared humidity term, and a temperature–humidity interaction feature. These derived variables were introduced to provide the RNN and LSTM models with additional nonlinear and physically interpretable predictors related to PV power variability, which is consistent with the broader deep-learning forecasting literature^10,14^.

7 $$\begin{array}{cccc}&\Delta T={T}_{component}-{T}_{ambient}& & \end{array}$$

8 $$\begin{array}{cccc}& {\eta}_{rad}=\frac{{P}_{actual}}{{G}_{total}},{G}_{total}>0& & \end{array}$$

9 $$\begin{array}{cccc}& {R}_{dir}=\frac{{G}_{direct}}{{G}_{total}},{G}_{total}>0& & \end{array}$$

10 $$\begin{array}{cccc}& {R}_{diff}=\frac{{G}_{diffuse}}{{G}_{total}},{G}_{total}>0& & \end{array}$$

11 $$\begin{array}{cccc}& {P}_{norm}=\frac{{P}_{atm}}{1013.25}& & \end{array}$$

12 $$\begin{array}{cccc}& {H}_{sq}={H}^{2}& & \end{array}$$

13 $$\begin{array}{cccc}& T{H}_{int}={T}_{ambient}\times H& & \end{array}$$

where $\:{\Delta\:}T$denotes the temperature difference between module and ambient temperatures, $\:{\eta\:}_{rad}$is the radiation-efficiency indicator, $\:{R}_{dir}$and $\:{R}_{diff}$represent the direct-to-total and diffuse-to-total radiation ratios, respectively, $\:{P}_{norm}$denotes the normalized atmospheric pressure, $\:{H}_{sq}$is the squared humidity term, $\:T{H}_{int}$is the temperature–humidity interaction feature, $\:H$is the relative humidity, and $\:{T}_{ambient}$is the ambient temperature.

**Fig. 2:**
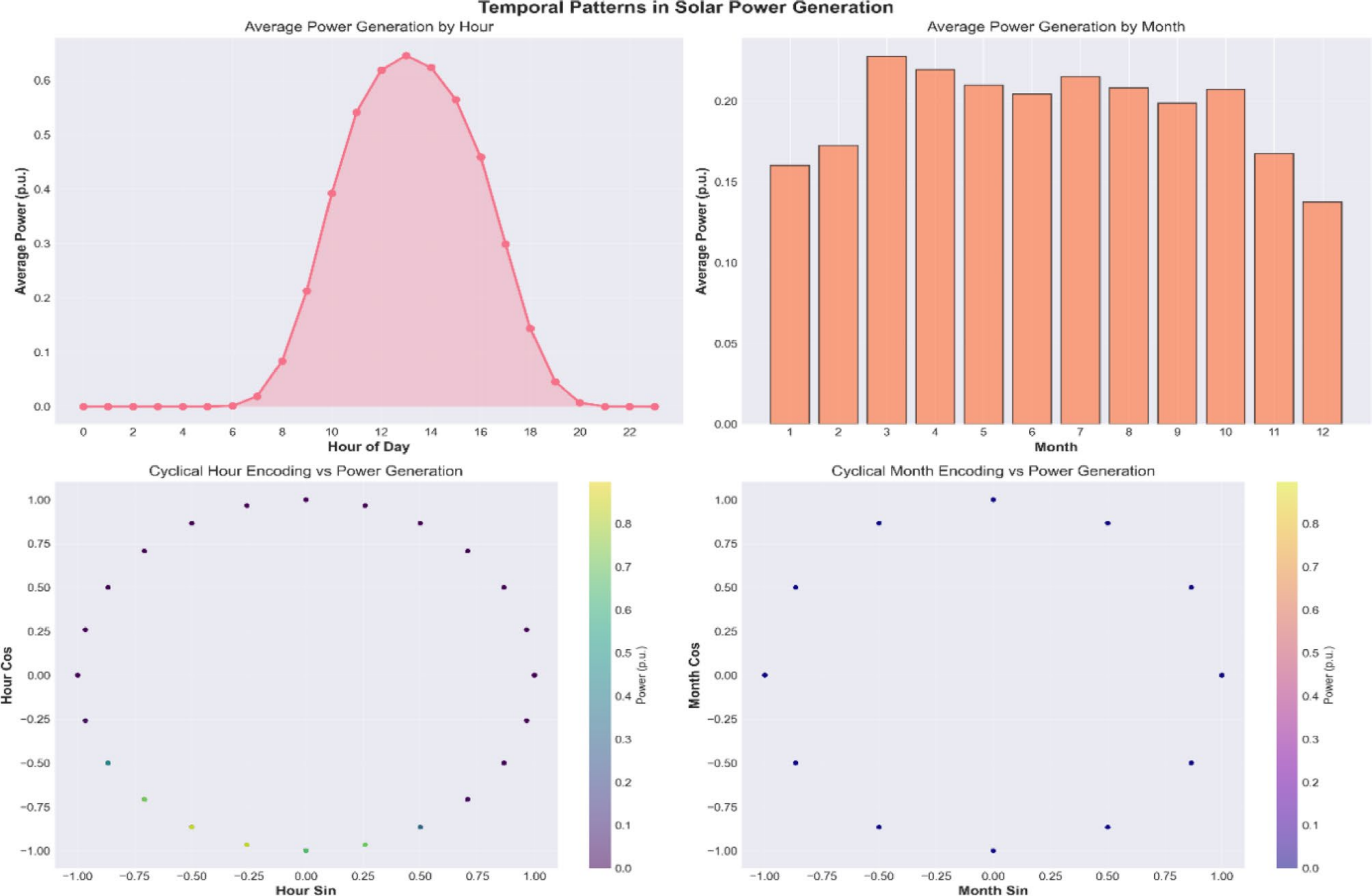
Temporal patterns in solar PV generation, including hourly and monthly average profiles and cyclical representations of time-related variables.

The temporal and statistical characteristics of the dataset were examined prior to model development. Figure 2 illustrates the hourly and monthly generation patterns together with the cyclical representation of time-related variables, highlighting the strong daytime production peak and the seasonal variation in PV output.

**Fig. 3:**
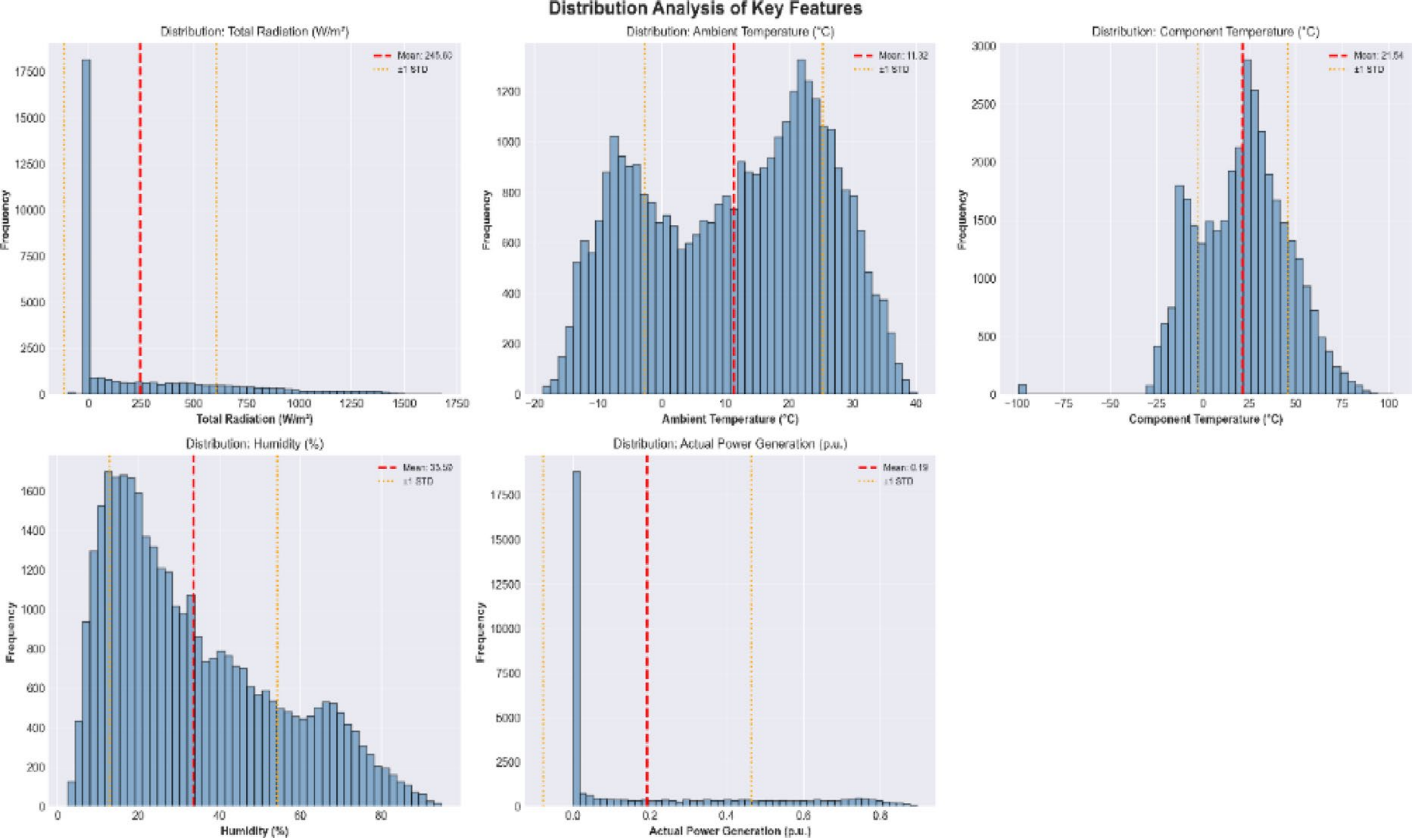
Empirical distributions of representative solar variables, including radiation, temperature, humidity, and PV power output.

Figure 3 summarizes the empirical distributions of representative meteorological variables and the target PV output. The irradiance-related variables exhibit strong non-Gaussian behavior due to the diurnal cycle, while humidity shows a positively skewed distribution. The PV power output is concentrated near zero during nighttime and becomes right-skewed during daytime operation. These characteristics justify the use of robust preprocessing and rank-based feature analysis in the subsequent stages.

#### Data cleaning, feature selection, and scaling

**Fig. 4:**
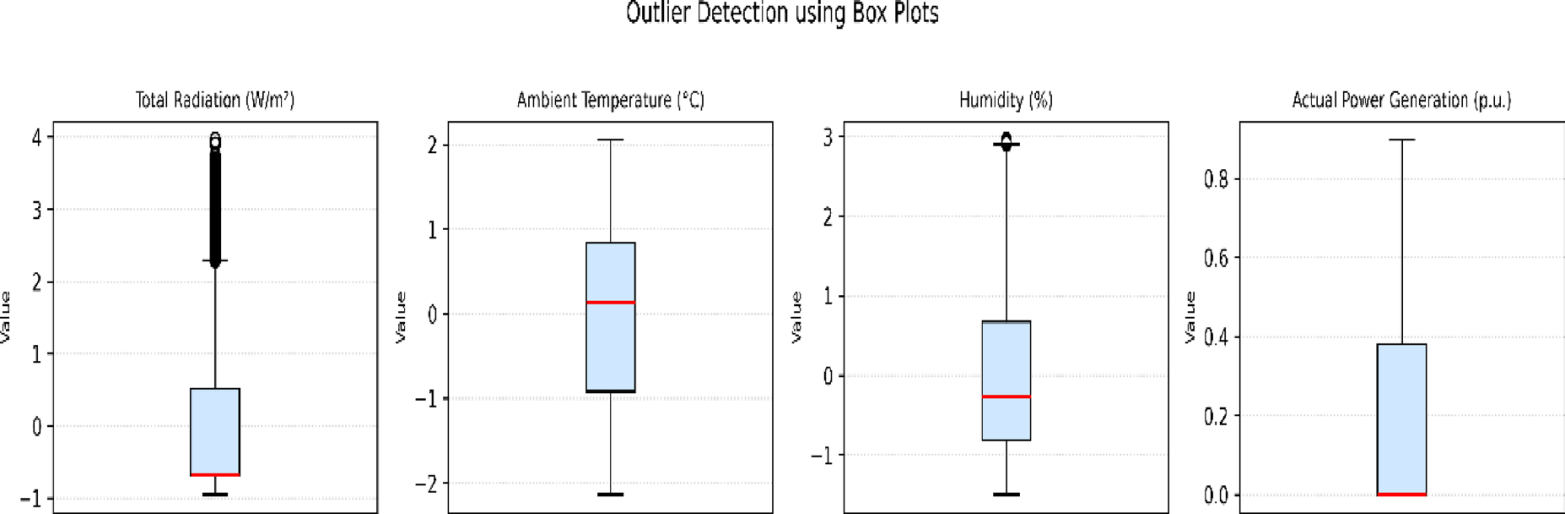
Box-plot diagnostics of representative meteorological variables and PV power output based on the interquartile-range (IQR) criterion.

The preprocessing pipeline explicitly checks for missing values, non-finite entries, and anomalous observations. Missing numerical values were handled using median imputation, and any positive or negative infinite values were first replaced and then imputed using the same strategy. In Fig. 4, Outliers were screened using the interquartile-range (IQR) criterion as a diagnostic data-quality step rather than an aggressive deletion rule, since extreme irradiance and PV-output values may still correspond to physically plausible operating conditions. This conservative treatment is suitable for solar forecasting data, where sharp meteorological transitions and high-irradiance events are not necessarily erroneous observations, and it is consistent with the statistical data-preparation principles^19^.

**Fig. 5:**
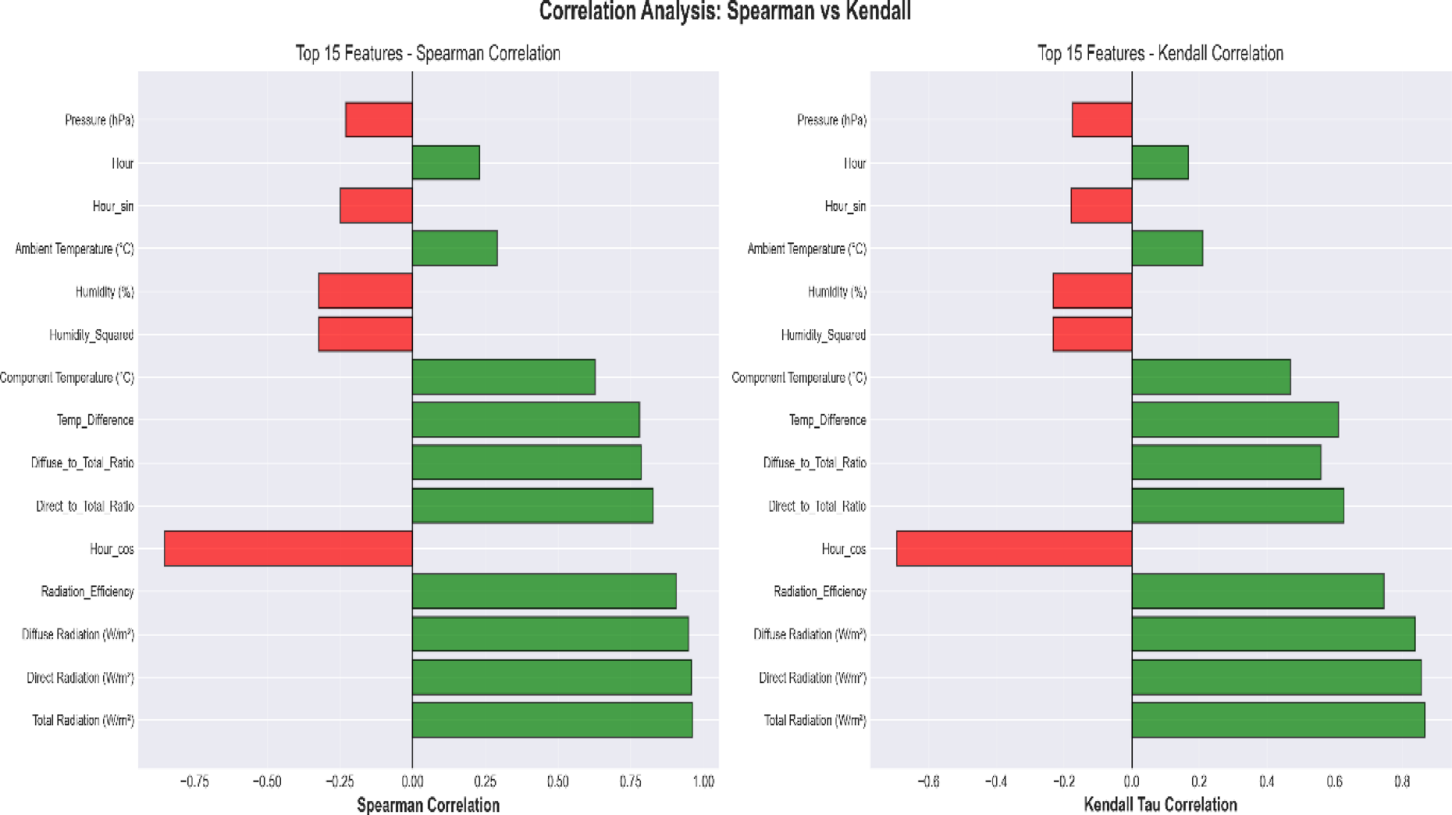
Rank-based feature relevance analysis using Spearman’s rho and Kendall’s tau.

Feature relevance was then assessed using two nonparametric rank-based measures, namely Spearman’s rho and Kendall’s tau, with respect to the target variable. This approach captures monotonic relationships while reducing sensitivity to non-Gaussian distributions and extreme values. As illustrated in Fig. 5, total radiation and direct radiation exhibit the strongest positive associations with PV generation, whereas nighttime-related indicators and humidity-related features show negative or weak associations.

Spearman’s rho and Kendall’s tau are computed as follows:

14 $${\rho}_{s}=1-\frac{6\sum_{\mathrm{i}=1}^{\mathrm{N}} {d}_{i}^{2}}{n\left({n}^{2}-1\right)}$$

Where $\:{d}_{i}$is the difference between the ranks of corresponding variables.

Kendall’s Tau is defined as:

15 $$\tau =\frac{{n}_{c}-{n}_{d}}{\frac{1}{2}n\left(n-1\right)}$$

Where $\:{n}_{c}$is the number of concordant pairs and $\:{n}_{d}$is the number of discordant pairs.

**Fig. 6:**
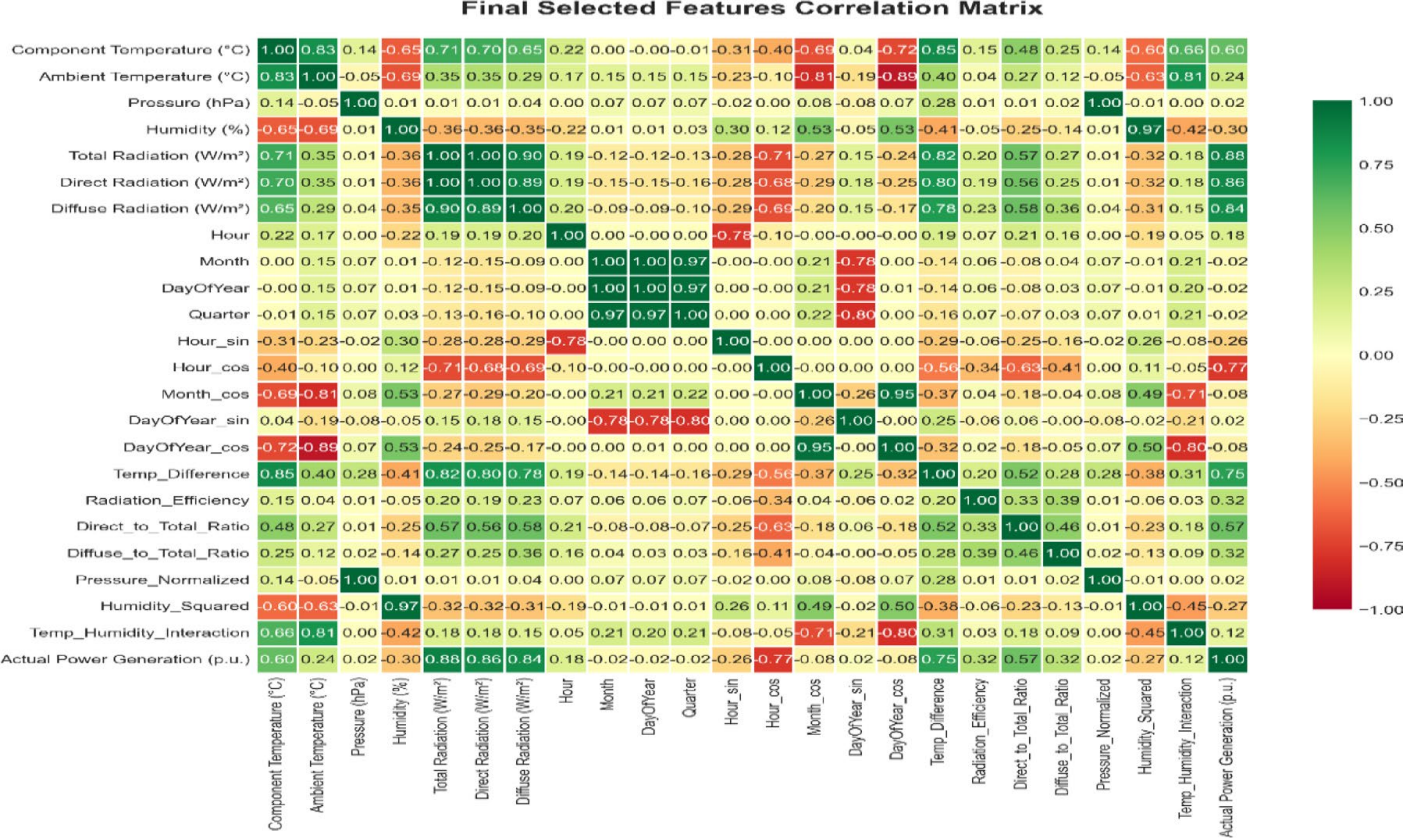
Correlation heatmap of the final selected features used for photovoltaic power forecasting.

In the implemented pipeline, the final feature subset was selected using the joint significance criterion of the two rank-based analyses. The resulting selected-feature correlation structure is shown in Fig. 6. The final feature set preserves the most informative predictors while reducing redundancy in the input space.

After feature construction and feature selection, the retained predictor variables were standardized using z-score normalization to improve numerical conditioning during optimization and gradient-based training. For a feature x, the standardized value z is given by:

16 $$\begin{array}{cccc}& z=\frac{x-\mu }{\sigma }& & \end{array}$$

where $\:x\:$represents the raw input feature, $\:\mu\:\:$denotes the mean of that feature, $\:\sigma\:\:$denotes its standard deviation, and $\:z$is the standardized feature value.

This scaling step ensures that variables expressed in different physical units contribute on comparable numerical scales during model training. The target variable, Actual Power Generation, was preserved in its original per-unit (p.u.) scale to maintain the physical interpretability of the forecasting outputs.

### Sequence generation

The forecasting task was formulated as a supervised sequence-to-one learning problem in which each input sample consists of a fixed-length multivariate history and the target corresponds to the PV power output at the next time step. Such a formulation is common in short-term PV forecasting because it enables recurrent models to exploit temporal dependence and recent persistence behavior in the observed series^20,21^.

A fixed lookback horizon of L = 10 time steps was used in the present implementation. Because the processed dataset was sampled at 15-minute intervals, this corresponds to a historical window of 2.5 h. Thus, at each time step t, the model receives the most recent L observations of the predictor variables and estimates the photovoltaic power output at time t + 1.

17 $$\begin{array}{cccc}& {X}_{t}=\left[{x}_{t-L+1},{x}_{t-L+2},\dots ,{x}_{t}\right],L=10& & \end{array}$$

18 $$\begin{array}{cccc}& {y}_{t}={P}_{t+1}& & \end{array}$$

where $\:{X}_{t\:\:}$denotes the multivariate input sequence at time step $\:t$, $\:{x}_{t}\:$is the feature vector at time step $\:t$, $\:L\:$is the lookback horizon, and $\:{y}_{t}$is the target output. This fixed-length sliding-window representation was used consistently for both the RNN and LSTM models.

**Fig. 7:**
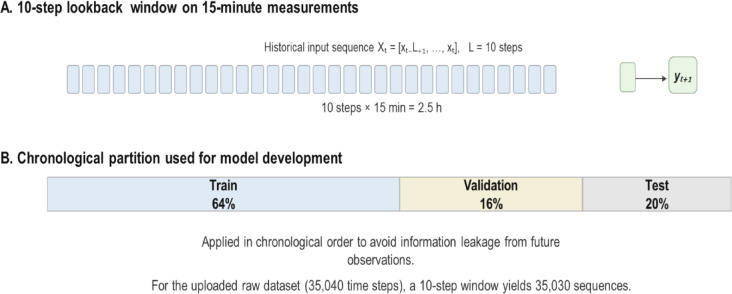
Sequence generation using a 10-step lookback horizon (2.5 h) and chronological data partitioning.

Figure 7 illustrates the sequence-generation mechanism and the subsequent data-partitioning workflow used for model development.

### Forecasting models

**Fig. 8:**
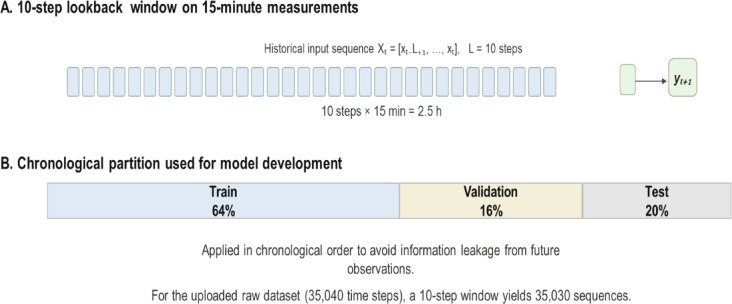
Generic stacked recurrent architecture is used for the forecasting models.

Two recurrent architectures are evaluated in this study: a standard Recurrent Neural Network (RNN) and a Long Short-Term Memory (LSTM) network^22,23^. Both models are implemented as stacked architectures composed of two recurrent layers, two dropout layers, and a dense output layer^24,25^. This configuration enables direct comparison between a simpler recurrent baseline and a gated recurrent alternative under identical optimization settings (Fig. 8).

For the standard RNN, the hidden state at time step $\:t$is computed recursively from the current input vector $\:{x}_{t}$and the previous hidden state $\:{h}_{t-1}$^22^:

19 $$\begin{array}{cccc}& {h}_{t}=\phi \left({W}_{xh}{x}_{t}+{W}_{hh}{h}_{t-1}+{b}_{h}\right)& & \end{array}$$

20 $$\begin{array}{cccc}& {\widehat{y}}_{t}={W}_{hy}{h}_{t}+{b}_{y}& & \end{array}$$

where $\:{x}_{t}$is the input vector at time step $\:t$, $\:{h}_{t-1}$and $\:{h}_{t}$denote the previous and current hidden states, $\:{W}_{xh}$and $\:{W}_{hh}$are trainable weight matrices, $\:{b}_{h}$is the bias term, and $\:\phi\:(\cdot\:)$is the activation function. Moreover, $\:{W}_{hy}$and $\:{b}_{y}$are the output-layer parameters, and $\:{\widehat{y}}_{t}$denotes the predicted photovoltaic power output. Equation (19) updates the hidden state using the current input and the previous hidden state, whereas Eq. (20) maps the hidden representation to the output prediction.

The LSTM extends this formulation by introducing gating mechanisms that regulate memory retention, information update, and output generation^23^:

21 $$\begin{array}{cccc}& {f}_{t}=\sigma \left({W}_{f}\left[{h}_{t-1},{x}_{t}\right]+{b}_{f}\right)& & \end{array}$$

22 $$\begin{array}{cccc}& {i}_{t}=\sigma \left({W}_{i}\left[{h}_{t-1},{x}_{t}\right]+{b}_{i}\right)& & \end{array}$$

23 $$\begin{array}{cccc}& {g}_{t}=\mathrm{tanh}\left({W}_{g}\left[{h}_{t-1},{x}_{t}\right]+{b}_{g}\right)& & \end{array}$$

24 $$\begin{array}{cccc}& {o}_{t}=\sigma \left({W}_{o}\left[{h}_{t-1},{x}_{t}\right]+{b}_{o}\right)& & \end{array}$$

25 $$\begin{array}{cccc}& {c}_{t}={f}_{t}\odot {c}_{t-1}+{i}_{t}\odot {g}_{t}& & \end{array}$$

26 $$\begin{array}{cccc}& {h}_{t}={o}_{t}\odot \mathrm{t}\mathrm{a}\mathrm{n}\mathrm{h}({c}_{t})& & \end{array}$$

where $\:{f}_{t}$, $\:{i}_{t}$, and $\:{o}_{t}$denote the forget, input, and output gates, respectively, $\:{g}_{t}$is the candidate cell state, $\:{c}_{t}$is the cell state, and $\:{h}_{t}$is the hidden state. The operator $\:\sigma\:(\cdot\:)$denotes the sigmoid activation, whereas $\:\mathrm{t}\mathrm{a}\mathrm{n}\mathrm{h}(\cdot\:)$denotes the hyperbolic tangent activation. This gated structure enables the LSTM to preserve and update temporal information more effectively than the standard RNN^23^.

### Hyperparameter optimization

Hyperparameter optimization in the present study was carried out using four strategies: a fixed manual baseline, Random Search, the canonical Grey Wolf Optimizer (GWO), and the proposed Stochastic Grey Wolf Optimizer (SGWO). The manual baseline corresponds to a single expert-selected configuration and therefore does not involve iterative search. Random Search was included as a tuner-based baseline because it provides a competitive non-exhaustive comparison and has been shown to be effective for hyperparameter optimization in high-dimensional spaces^7^. In the implemented experiments, the wolf-based optimizers (GWO and SGWO) explored a six-dimensional mixed continuous–integer search space, whereas Random Search was applied to a five-parameter subspace with a fixed batch size of 32. This distinction reflects the experimental protocol and is reported to ensure methodological reproducibility.

For the wolf-based optimizers, each candidate solution is represented by a position vector

27 $${\mathbf{x}}_{i}=[{x}_{i,1},{x}_{i,2},{x}_{i,3},{x}_{i,4},{x}_{i,5},{x}_{i,6}]\in {\mathbb{R}}^{6}$$

which is decoded into the hyperparameter vector

28 $${{\boldsymbol{\theta}}}_{i}=\left[{u}_{1},{u}_{2},{d}_{1},{d}_{2},\eta ,b\right]$$

Here, $\:{u}_{1}$and $\:{u}_{2}$denote the numbers of units in the first and second recurrent layers, $\:{d}_{1}$and $\:{d}_{2}$are the corresponding dropout rates, $\:\eta\:$is the Adam learning rate, and $\:b$is the batch size. After clipping each coordinate to its feasible range, the integer-valued parameters are obtained as

29 $$u_{1} = x_{i,1} ,u_{2} = x_{i,2} ,b = x_{i,6}$$

whereas the continuous-valued parameters remain unchanged:

30 $${d}_{1}={x}_{i,3},{d}_{2}={x}_{i,4},\eta ={x}_{i,5}$$

Accordingly, the search space is explicitly mixed: the layer sizes and batch size are handled as discrete variables after decoding, whereas the dropout rates and learning rate are treated as continuous variables. The ranges of all hyperparameters are summarized in Table 3. In all experiments, the optimizer type was fixed to Adam, while the learning rate was treated as a tunable hyperparameter during model selection and then kept fixed within the corresponding training run^26^. Thus, the learning rate varied across candidate configurations during hyperparameter search, but it did not change epoch by epoch within an individual training run.

Each candidate configuration was evaluated through the training routine described in Section F, and the optimization objective was defined as the validation mean absolute error:

31 $$f\left({{\boldsymbol{\theta}}}_{i}\right)={\mathrm{M}\mathrm{A}\mathrm{E}}_{\mathrm{v}\mathrm{a}\mathrm{l}}\left({{\boldsymbol{\theta}}}_{i}\right)$$

The configuration achieving the minimum validation MAE was retained as the best hyperparameter setting for the corresponding model class.

**Table 3:** Hyperparameter search space used for RNN and LSTM tuning.

Hyperparameter	Type	Range	Description
units_1_	Integer	[10, 100]	Units in the first recurrent layer
units_2_	Integer	[10, 100]	Units in the second recurrent layer
dropout_1_	Real	[0.1, 0.5]	Dropout after recurrent layer 1
dropout_2_	Real	[0.1, 0.5]	Dropout after recurrent layer 2 / before output
learning rate	Real	[0.0001, 0.01]	Adam learning rate
batch size	Integer	[16, 64]	Mini-batch size during training

#### Grey Wolf Optimizer (GWO)

In the canonical GWO, the three best candidate solutions at iteration $\:t$are denoted by the leader positions $\:{\boldsymbol{\alpha\:}}^{\left(t\right)}$, $\:{\boldsymbol{\beta\:}}^{\left(t\right)}$, and $\:{\boldsymbol{\delta\:}}^{\left(t\right)}$, and the remaining wolves update their positions relative to these leaders (Mirjalili, Mirjalili, and Lewis 2014). For wolf $\:i$, dimension $\:j$, and leader index $\:k\in\:\{\mathrm{1,2},3\}$, the control coefficients are defined as.

32 $${A}_{k,j}^{\left(t\right)}=2{\hspace{0.17em}}{a}^{\left(t\right)}{\hspace{0.17em}}{r}_{1,k,j}^{\left(t\right)}-{a}^{\left(t\right)}$$

33 $${C}_{k,j}^{(t)}=2{\hspace{0.17em}}{r}_{2,k,j }^{(t)}$$

where $\:{r}_{1,k,j}^{\left(t\right)}$and $\:{r}_{2,k,j}^{\left(t\right)}$are independent random variables uniformly distributed in $\:\left[\mathrm{0,1}\right]$, and the linearly decreasing coefficient is given by

34 $${a}^{(t)}=2-2\frac{t}{T}$$

with $\:T$denoting the maximum number of iterations.

The distances from wolf $\:i$to the three leaders are then computed componentwise as.

35 $${D}_{\alpha ,j}^{\left(t\right)}=\mid {C}_{1,j}^{\left(t\right)}{\hspace{0.17em}}{\alpha}_{j}^{\left(t\right)}-{x}_{i,j}^{\left(t\right)}\mid$$

36 $${D}_{\beta ,j}^{\left(t\right)}=\mid {C}_{2,j}^{\left(t\right)}{\hspace{0.17em}}{\beta}_{j}^{\left(t\right)}-{x}_{i,j}^{\left(t\right)}\mid$$

37 $${D}_{\delta ,j}^{(t)}=\mid {C}_{3,j}^{\left(t\right)}{\hspace{0.17em}}{\delta}_{j}^{\left(t\right)}-{x}_{i,j}^{\left(t\right)}\mid$$

The leader-guided candidate coordinates are

38 $${X}_{1,j}^{\left(t\right)}={\alpha}_{j}^{\left(t\right)}-{A}_{1,j}^{\left(t\right)}{D}_{\alpha ,j}^{\left(t\right)}$$

39 $${X}_{2,j}^{\left(t\right)}={\beta}_{j}^{\left(t\right)}-{A}_{2,j}^{\left(t\right)}$$

40 $${X}_{3,j}^{\left(t\right)}={\delta}_{j}^{\left(t\right)}-{A}_{3,j}^{\left(t\right)}{D}_{\delta ,j}^{\left(t\right)}$$

The updated coordinate is then obtained as the mean of the three leader-guided components:

41 $${x}_{i,j}^{\left(t+1\right)}=\frac{{X}_{1,j}^{\left(t\right)}+{X}_{2,j}^{\left(t\right)}+{X}_{3,j}^{\left(t\right)}}{3}$$

This update rule is computationally simple and often effective, but its deterministic structure may reduce population diversity and increase the risk of premature convergence in rugged optimization landscapes^9,11,12^.

#### Stochastic Grey Wolf Optimizer (SGWO)

To improve exploration, the implemented SGWO modifies the canonical GWO update by injecting independent Gaussian perturbations into the three leader-guided components before averaging. The following formulation describes the SGWO update rule used in the present experiments. For each wolf $\:i$, dimension $\:j$, iteration $\:t$, and leader-guided component $\:k\in\:\{\mathrm{1,2},3\}$, let

42 $${\xi}_{k,j}^{\left(t\right)}\sim \mathcal{N}\left(\mathrm{0,1}\right)$$

be an independent Gaussian random variable generated at each iteration and for each dimension. With a fixed perturbation coefficient $\:\sigma\:$, the stochastic leader-guided coordinates are computed as.

43 $${X}_{1,j}^{\left(t\right)}={\alpha}_{j}^{\left(t\right)}-{A}_{1,j}^{\left(t\right)}{D}_{\alpha ,j}^{\left(t\right)}+\sigma {\hspace{0.17em}}{\xi}_{1,j}^{\left(t\right)}{\hspace{0.17em}}{\alpha}_{j}^{\left(t\right)}$$

44 $${X}_{2,j}^{\left(t\right)}={\beta}_{j}^{\left(t\right)}-{A}_{2,j}^{\left(t\right)}{D}_{\beta ,j}^{\left(t\right)}+\sigma {\hspace{0.17em}}{\xi}_{2,j}^{\left(t\right)}{\hspace{0.17em}}{\beta}_{j}^{\left(t\right)}$$

45 $${X}_{3,j}^{\left(t\right)}={\delta}_{j}^{\left(t\right)}-{A}_{3,j}^{\left(t\right)}{D}_{\delta ,j}^{\left(t\right)}+\sigma {\hspace{0.17em}}{\xi}_{3,j}^{\left(t\right)}{\hspace{0.17em}}{\delta}_{j}^{\left(t\right)}$$

The SGWO position update is therefore

46 $${x}_{i,j}^{(t+1)}=\frac{{X}_{1,j}^{(t)}+{X}_{2,j}^{(t)}+{X}_{3,j}^{(t)}}{3}$$

In the implemented experiments, the perturbation coefficient was fixed at $\:\sigma\:=0.1\:\:$for all iterations. Hence, the stochastic term was not annealed during the run; instead, diversity was introduced through fresh Gaussian perturbations generated independently for each leader contribution, each dimension, and each iteration. This design was adopted to improve exploration and reduce premature convergence while preserving the basic leader-guided structure of the original GWO^11,12^.

After each update, the candidate position vector is clipped component wise to the feasible bounds before decoding. Integer-valued hyperparameters, namely $\:{u}_{1}$, $\:{u}_{2}$, and $\:b$, are then obtained by integer casting after clipping, while the dropout rates and learning rate remain continuous. The best-performing candidate across the search process is ultimately selected for the corresponding RNN or LSTM model. Across all optimization strategies, the fitness function is defined as the validation MAE produced by the model training routine. The configuration yielding the lowest validation MAE is retained as the optimal hyperparameter setting and subsequently used for final model evaluation.

**Algorithm 1:**
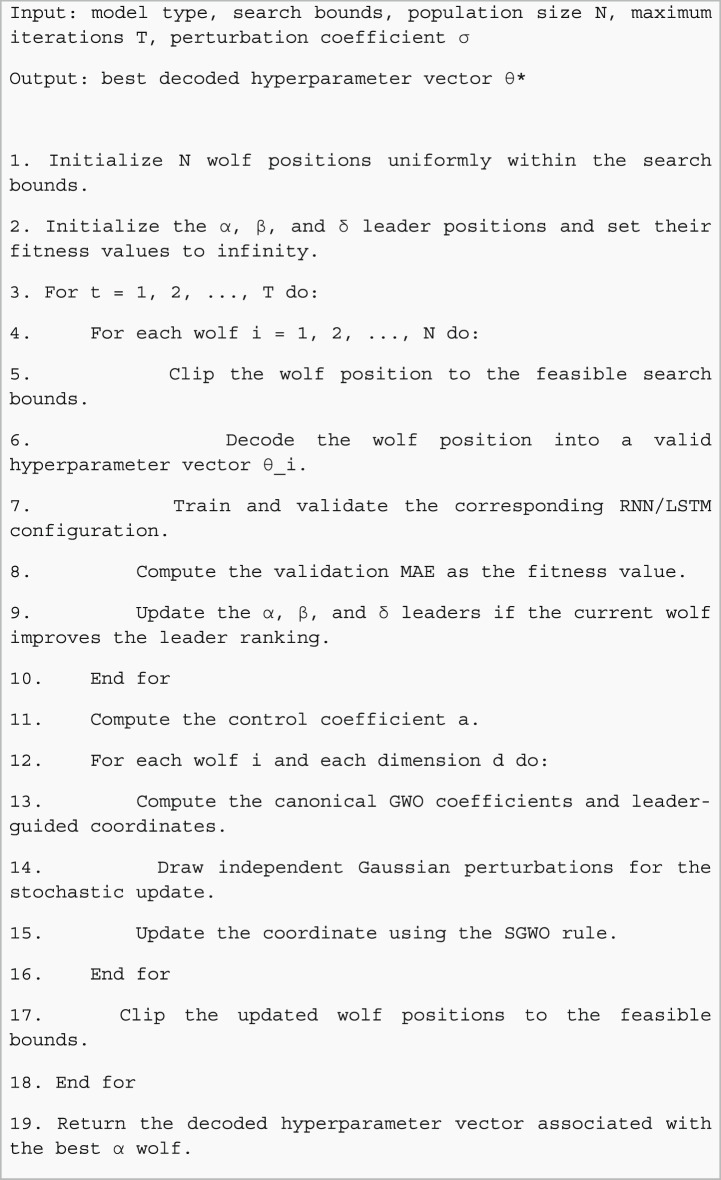
SGWO-based hyperparameter optimization used in this study.

The computational complexity of the implemented SGWO procedure can be expressed as follows:

$$\:{C}_{SGWO}\:=\:O(TN{C}_{train}\:+\:TNd)$$

where T denotes the number of SGWO iterations, N denotes the number of wolves, d denotes the dimensionality of the hyperparameter search space, and C_train denotes the computational cost of training and validating one candidate RNN/LSTM configuration.

The two terms in the complexity expression correspond to:

$$\:{C}_{eval}\:=\:O\left(TN{C}_{train}\right)$$

$$\:{C}_{update}\:=\:O\left(TNd\right)$$

Thus, the overall complexity is:

$$\:{C}_{SGWO}\:=\:{C}_{eval}\:+\:{C}_{update}\:=\:O(TN{C}_{train}\:+\:TNd)$$

Since model training is computationally more expensive than the SGWO coordinate-update operations, the practical complexity can be approximated as:

$$\:{C}_{SGWO}\:\approx\:\:O\left(TN{C}_{train}\right)$$

In the present implementation:

$$\:T\:=\:5,\:\:\:N\:=\:5,\:\:\:d\:=\:6$$

Therefore, the number of candidate evaluations during the SGWO search is:

$$\:T\:\times\:\:N\:=\:5\:\times\:\:5\:=\:25$$

Accordingly, the SGWO search performs 25 candidate model evaluations, followed by one final training run using the best selected hyperparameter configuration (Table 4).

**Table 4:** SGWO configuration used in the study.

Parameter	Symbol	Value	Description
Population size	$\:{n}_{\mathrm{wolves}}$	5	Number of wolves in the search population
Iterations	$\:\mathrm{T}$	5	Number of optimization iterations
Noise coefficient	$\:\sigma\:$	0.1	Gaussian perturbation coefficient
Objective function	–	Validation MAE	Fitness returned by the training routine
Bounds	$\:lb,ub$	Table 3	Lower and upper bounds of the search space

The hyperparameter configuration yielding the lowest validation MAE was retained for final model training and evaluation.

### Training and validation protocol

The model training was done in one single optimizing procedure to enable comparisons among the three different configurations (Baseline, Random Search, GWO, SGWO). All experiments employed the Adam Optimizer to optimize the weights of the network. However, the Learning Rate, that was searched over as part of the Hyper-Parameter search in Section E and thus remained constant throughout each training run, was selected through this process. Training Loss was measured as Mean Absolute Error (MAE) while Mean Squared Error (MSE) was tracked as an additional performance measure for tracking convergence during training^26^.

The supervised sequences generated by the model with a fixed look back window of length L = 10 (which corresponds to 2.5 h based on the 15 min sampling period) were divided using a hold out approach. A window size of L = 10 was chosen to provide a relatively small short term time series context that would capture some degree of recent trend persistence and intra-day dependence in PV output generation, while also keeping the number of inputs and computational complexity associated with training as low as possible. Additionally, the window length was held constant for all compared models/optimization strategies to maintain methodological consistency^13,20^.

In addition, to prevent temporal dependencies from being compromised at the final evaluation step, 20% of the generated sequences were set aside and used as a blind test subset using a non-shuffled split. The remaining 80%, which formed the development subset used for selecting models/hyperparameters, was then split into training and validation subsets using a fixed random seed. Overall, this resulted in a split that allocated approximately 64% for training, 16% for validating and 20% for testing. This configuration preserves both the integrity of the final test results and maintains a separate validation subset for controlling early stopping and hyper-parameter tuning, which are typical practices when evaluating sequential data^27,28^.

Let $\:N$denote the total number of generated supervised sequences. The subset sizes are given by

47 $${N}_{\mathrm{t}\mathrm{e}\mathrm{s}\mathrm{t}}=0.20N,{N}_{\mathrm{d}\mathrm{e}\mathrm{v}}=0.80N,{N}_{\mathrm{v}\mathrm{a}\mathrm{l}}=0.20{N}_{\mathrm{d}\mathrm{e}\mathrm{v}},{N}_{\mathrm{t}\mathrm{r}\mathrm{a}\mathrm{i}\mathrm{n}}=N-{N}_{\mathrm{t}\mathrm{e}\mathrm{s}\mathrm{t}}-{N}_{\mathrm{v}\mathrm{a}\mathrm{l}}$$

In the present implementation, the processed dataset of 35,040 observations yielded 35,030 supervised sequences after applying the 10-step sliding window. Accordingly, the final partition consisted of 22,419 training sequences, 5,605 validation sequences, and 7,006 test sequences, as summarized in Table 5.

**Table 5:** Final data partitioning used after sequence generation.

Subset	Basis of split	Share of all generated sequences	Number of sequences
Training set	Remaining sequences within the development subset after validation allocation	64.0%	22,419
Validation set	20% of the development subset	16.0%	5,605
Test set	Final 20% of the generated sequences reserved as an unseen hold-out subset	20.0%	7,006
Total	All generated sequences	100.0%	35,030

For each model search, all candidate configurations were tested on the same evaluation protocol. The best performing configuration based on the lowest validation Mean Absolute Error (MAE) was selected for that particular model search. Early stopping is used to determine if the model has reached its optimal performance or if it will continue to overfit the data. Validation loss is monitored throughout each training session. If the validation loss does not decrease after three consecutive evaluations (patience = 3), the model will stop evaluating and save the best weights from that point. To increase reproducibility of results in this project, fixed random seed values were set in both Python, NumPy, and TensorFlow. Additionally, multiple max epochs were evaluated during experimentation (E = 5, 10, 50, 100, and 150). However, depending on whether the validation loss stopped decreasing prior to reaching the maximum number of epochs, fewer than the specified max epochs may have actually been completed.

A single primary experimental environment utilized a maximum of 5 epochs for an overall budget to maintain computational feasibility and allow for comparison amongst each model-optimizer combination. In order to utilize this fixed primary evaluation protocol, it was not to be considered that 5 epochs was a universal best number of epochs for all types of models and training paradigms. Additional experiments were performed to evaluate the sensitivity of our results to increasing numbers of iterations, with the results from these increased numbers of epochs (i.e., 10, 50, 100, 150) presented in Appendix A and discussed in Section IV.C.

Rolling-origin evaluation and conventional k-fold cross-validation were not used in this study. Instead, all model comparisons followed the same fixed hold-out protocol, so that the baseline, Random Search, GWO, and SGWO strategies were evaluated using identical validation criteria and test partitions. Within each training run, the learning rate was fixed after hyperparameter selection and did not vary from epoch to epoch.

The final predictive performance of the selected configurations was then evaluated on the untouched test subset using the regression metrics defined in Section G.

### Performance evaluation metrics

Forecasting performance was evaluated using four standard regression metrics: root mean square error (RMSE), mean absolute error (MAE), mean squared error (MSE), and the coefficient of determination ($\:{R}^{2}$). These metrics are widely used in forecasting and machine-learning studies because they provide complementary information about prediction accuracy, average error magnitude, sensitivity to large deviations, and explanatory power^29–31^.In particular, MAE provides a direct and scale-preserving measure of the average absolute forecasting error, whereas MSE and RMSE assign greater weight to larger deviations because they are based on squared errors. The coefficient of determination ($\:{R}^{2}$) quantifies the proportion of variance in the observed data explained by the model predictions.

The root mean square error is defined as

48 $$\mathrm{R}\mathrm{M}\mathrm{S}\mathrm{E}=\sqrt{\frac{1}{N}\sum_{i=1}^{N}({y}_{i}-{\widehat{y}}_{i}{)}^{2}}$$

where $\:N$is the number of observations in the evaluation set, $\:{y}_{i}$is the actual observed value for sample $\:i$, and $\:{\widehat{y}}_{i}$is the corresponding model prediction.

The mean absolute error is defined as

49 $$\mathrm{M}\mathrm{A}\mathrm{E}=\frac{1}{N}\sum_{i=1}^{N}\mid {y}_{i}-{\widehat{y}}_{i}\mid$$

where $\:N$denotes the total number of evaluated samples, $\:{y}_{i}$represents the true observed value, and $\:{\widehat{y}}_{i}$denotes the predicted value produced by the forecasting model.

The mean squared error is defined as

50 $$\mathrm{M}\mathrm{S}\mathrm{E}=\frac{1}{N}\sum_{i=1}^{N}({y}_{i}-{\widehat{y}}_{i}{)}^{2}$$

In the present study, MSE was additionally reported to provide a squared-error view of predictive performance and was obtained directly from the corresponding test-set RMSE values through the identity $\:\mathrm{M}\mathrm{S}\mathrm{E}={\mathrm{R}\mathrm{M}\mathrm{S}\mathrm{E}}^{2}$

The coefficient of determination is computed as

51 $$R^{2} = 1 - \frac{{\mathop \sum \nolimits_{{i = 1}}^{N} (y_{i} - \hat{y}_{i} )^{2} }}{{\mathop \sum \nolimits_{{i = 1}}^{N} (y_{i} - \bar{y})^{2} }}$$

where $\:{y}_{i}$is the observed value for sample $\:i$, $\:{\widehat{y}}_{i}$is the corresponding predicted value, $\bar{y}$is the mean of the observed values in the evaluation set, and $\:N$is the total number of samples.

These metrics were used consistently to compare the forecasting performance of all model–optimizer combinations considered in this study. MAE was used to quantify the average absolute prediction error, MSE and RMSE were used to emphasize larger deviations, and $\:{R}^{2}$was used to evaluate goodness of fit. Using these four metrics together provides a balanced and transparent evaluation framework for solar PV forecasting^29–31^.

Relative or percentage-based error metrics were not adopted in the main evaluation protocol. Such measures involve division by the observed value and may become unstable or undefined when the target series contains zero or near-zero values, which is a relevant issue in photovoltaic power forecasting because zero-output periods occur naturally during nighttime hours. For this reason, the present study relies on MAE, MSE, RMSE, and $\:{R}^{2}$, which remain well-defined and numerically stable under zero-valued observations^30,32^.

### Implementation environment

All preprocessing, sequence generation, model training, and hyperparameter optimization procedures were implemented in Python within a Jupyter Notebook environment. The workflow relied on widely used scientific-computing and deep-learning libraries to ensure reproducibility and methodological transparency throughout the study. In particular, pandas and NumPy were used for timestamp handling, tabular data manipulation, numerical operations, and sequence construction; SciPy was used for selected statistical analyses; and scikit-learn was used for preprocessing utilities, dataset partitioning, scaling, and evaluation-related functions. The recurrent forecasting models were implemented using TensorFlow/Keras, which provided the framework for defining and training the RNN and LSTM architectures. In addition, Matplotlib and Seaborn were used to generate the exploratory and diagnostic figures reported in this study.

All experiments were conducted on a Victus by HP Gaming Laptop 15-fa1xxx running Windows 11 (64-bit), equipped with a 12th Gen Intel^®^ Core™ i7-12650 H processor, 16 GB RAM, and a discrete GPU with approximately 6 GB VRAM. The computational environment was managed through Anaconda and executed in Jupyter Notebook. This hardware–software configuration was sufficient to support the repeated training and comparative evaluation of the baseline, random-search, GWO, and SGWO experiments under a consistent execution environment.

This subsection reports the computational environment and core software stack used to execute the experiments. Table 6 summarizes the principal software components used in the workflow.

**Table 6:** Core software components used in the workflow.

Library / framework	Role in the workflow	Version	URL
pandas	Timestamp parsing, tabular data handling, and sequence preparation	2.2.2	https://pandas.pydata.org/
NumPy	Numerical array operations and tensor reshaping	1.26.4	https://numpy.org/
SciPy	Correlation analysis and selected statistical functions	1.13.1	https://scipy.org/
scikit-learn	Dataset partitioning, preprocessing, scaling, and evaluation functions	1.7.1	https://scikit-learn.org/
TensorFlow / Keras	Definition and training of RNN/LSTM models	TF- 2.18.0 Keras- 3.8.0	https://www.tensorflow.org/ https://keras.io/
Matplotlib / Seaborn	Exploratory plots and model-diagnostic figures	Matplotlib- 3.9.2 Seaborn- 0.13.2	https://matplotlib.org https://seaborn.pydata.org

## Results and discussion

This section evaluates the proposed SGWO framework from both optimization and forecasting perspectives. First, SGWO is compared with the classical GWO on a suite of benchmark functions in order to assess its search behavior under controlled optimization settings. Next, the optimized hyperparameter configurations are evaluated on the photovoltaic forecasting task using RNN and LSTM architectures. Additional analyses of training budget, convergence behavior, and validation performance are then presented to examine robustness and generalization. Finally, the practical implications and the main limitations of the present study are discussed.

### Benchmark functions

The mathematical definitions, dimensions, and variable bounds of the test problems follow the standard global optimization set reported by^33^.

**Table 7:** Unimodal functions (F1–F5)^33^.

Function (ID & Name)	Dim	Range / Domain	Global optimum f_min_
F1: Sphere	30	$\:x\in\:\:\left[-100,\:100\right],\:\:i\:=\:1\:,30$	f_min_ = 0 at x^*^ = f(0,.,0)
F2: Ackley	30	$\:x\in\:$ [− 32.768, 32.768], $\:i\:=\:1\:,30$	f_min_ = 0 at x^*^ = f(0,…,0)
F3: Griewank	30	$\:x\in\:[-600,\:600$], $\:i\:=\:1\:,30$	f_min_ = 0 at x^*^ = f(0,…,0)
F4: Rastrigin	30	$\:x\in\:[-5.12,\:5.12],\:\:i\:=\:1\:,30$	f_min_ = 0 at x^*^ =f (0,…,0)
F5: Rosenbrock	30	$\:x\in\:[-30,\:30],\:\:i\:=\:1\:,30$	f_min_ = 0 at x^*^ = f(1,…,1)

**Table 8:** Multimodal functions (F6–F9)^33^.

Function (ID & Name)	Dim	Range / Domain	Global optimum f_min_
F6: Zakharov	30	$\:x\in\:[-5,\:10],\:i\:=\:1\:,30$	f_min_ = 0 at x^*^ = f(0,…,0)
F7: Levy	30	$\:x\in\:[-10,\:10],\:i\:=\:1\:,30$	f_min_ = 0 at x^*^ = f(1,…,1)
F8: Schwefel 2.26	30	$\:x\in\:[-500,\:500],\:i\:=\:1\:,30$	f_min_ ≈ − 418.9829 × 30 at $\:{x}_{i}^{*}$≈ 420.9687
F9: Dixon–Price	30	$\:x\in\:[-\mathrm{10,10}],\:i\:=\:1\:,30$	f_min_ = 0 at x^*^ with x_1_ = 1, $\:{x}_{i}={2}^{-\frac{{2}^{i}-\:2}{{2}^{i}}}$, i ≥ 2

**Table 9:** Penalized/other functions ( F10–F11 are adapted from^33,34^, whereas F12–F13^34^.

Function (ID & Name)	Dim	Range / Domain	Global optimum f_min_
F10: Schwefel 2.21	30	$\:x\in\:[-\mathrm{100,100}],\:i\:=\:1\:,30$	f_min_ = 0 at x^*^ = f(0,…,0)
F11: Schwefel 2.22	30	$\:x\in\:[-\mathrm{100,100}],\:\:i\:=\:1\:,30$	f_min_ = 0 at x^*^ = f(0,…,0)
F12: Penalized1	30	$\:x\in\:[-\mathrm{50,50}],\:\:i\:=\:1\:,30$	f_min_ = 0 at x^*^ =f (− 1,…,−1)
F13: Penalized2	30	$\:x\in\:[-\mathrm{50,50}],\:\:i\:=\:1\:,30$	f_min_ in = 0 at x^*^ = f(− 1,…,−1)

**Table 10:** Functions (F14–F19)^33^.

Function (ID & Name)	Dim	Range / Domain	Global optimum f_min_
F14: Shekel’s Foxholes	2	$\:{\mathrm{x}}_{1},\:{\mathrm{x}}_{2}\in\:\:[-65.536,\:65.536]$	f_min_ ≈ 1.0 (standard Shekel’s Foxholes definition)
F15: Kowalik	4	$\:x\in\:[-\mathrm{5,5}],\:\:i\:=\:1\:,4$	f_min_ ≈ 3.07 × 10^(−4)^
F16: Six-Hump Camel	2	$\:{x}_{1}\in\:\:\left[-\mathrm{3,3}\right],\:{x}_{2}\in\:\:\:\:[-\mathrm{2,2}]$	f_min_ ≈ − 1.0316
F17: Branin (Branin–Hoo)	2	$\:{x}_{1}\in\:\:\left[-\mathrm{5,10}\right],\:{x}_{2}\in\:\:\:\:\left[\mathrm{0,15}\right]$	f_min_ ≈ 0.3979
F18: Goldstein–Price	2	$\:{x}_{1},\:{\mathrm{x}}_{2}\in\:\:\:\:[-\mathrm{2,2}]$	f_min_ = 3 at x^*^ = (0, − 1)
F19: Hartmann3	3	$\:x\in\:\left[\mathrm{0,1}\right],\:i\:=\:1\:,2\:\:,3$	f_min_ ≈ − 3.8628

It is important to note that F1–F13 are tested in a 30-dimensional setting to evaluate the scalability of the optimizers on high-dimensional landscapes. In contrast, F14–F19 are classical fixed-dimension benchmarks (with Dim = 2, 3, or 4 as listed in Table 10) and are thus evaluated in their standard dimensionality as reported in^33^.

Tables 7, 8, 9 and 10. Overview of benchmark test functions (IDs, types, dimensions, domains, and global optima).

**Table 11:** Experimental settings and common parameterization across algorithms.

Alg.	Pop.	Max Iter	FE budget	Runs	Seeds	Init.	Boundary	Platform
{GWO, SGWO}	30	500	pop × iters	30	Fixed list	Uniform	Reflection	Win10/Python

**Table 12:**
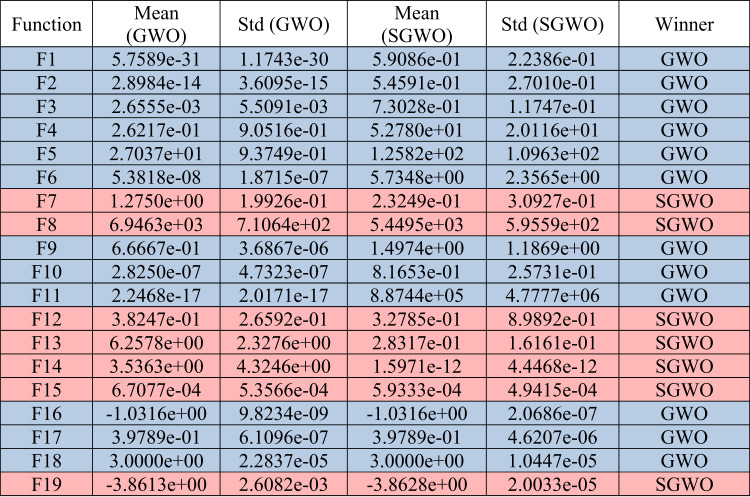
Aggregated performance per function (mean ± std over multiple runs). Best mean per row is shaded; lower is better.

Aggregated performance per function (mean ± std over multiple runs) is reported in Table 12, where lower values indicate better solutions. GWO attains lower mean objective values on the majority of the simpler, smooth functions, particularly the unimodal and less rugged cases such as F1–F6, F9, F10, F11, F16, F17, and F18. In contrast, SGWO achieves better performance on several multimodal and more challenging landscapes, namely F7, F8, F12, F13, F14, F15, and F19. This pattern confirms that GWO exhibits stronger exploitation and faster convergence on relatively well-behaved functions, whereas SGWO provides enhanced exploration and robustness on rugged, non-convex search spaces. The complementary strengths of the two optimizers are clearly visible in the per-function summary of Table 12.

#### Convergence figures

**Fig. 9:**
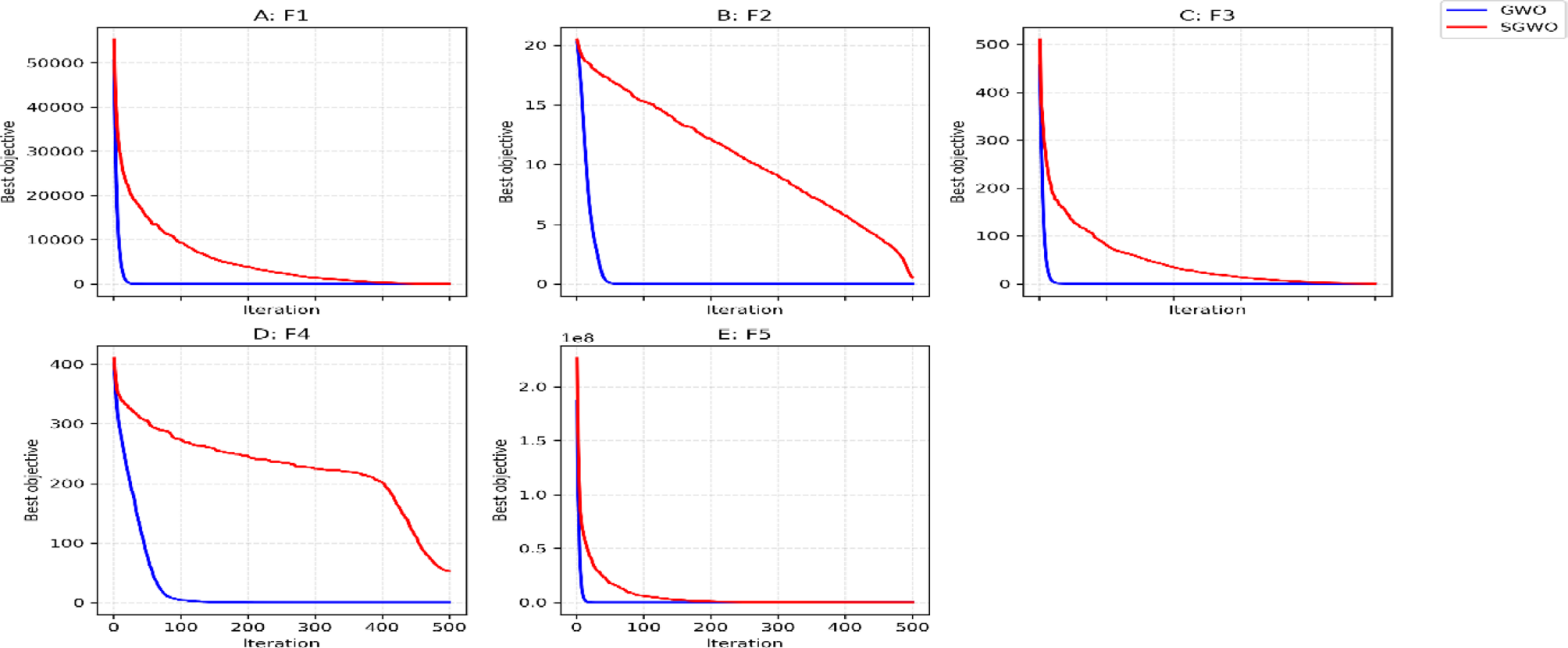
Convergence curves on F1–F5.

GWO consistently attains lower final objective values on all unimodal functions F1–F5, indicating stronger exploitation and faster convergence on smooth, well-behaved landscapes. SGWO converges more slowly and remains trapped at higher objective values on these functions.

**Fig. 10:**
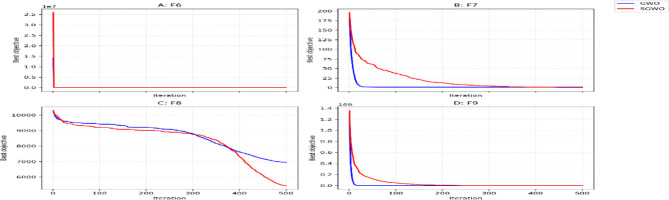
Convergence curves on F6–F9.

On F6 and F9, GWO converges to lower final objective values, reflecting more effective exploitation on these cases. In contrast, SGWO clearly outperforms GWO on F7 and F8 by reaching substantially lower objective values and maintaining smaller variability across runs, which highlights SGWO’s improved exploratory behavior on multimodal landscapes.

**Fig. 11:**
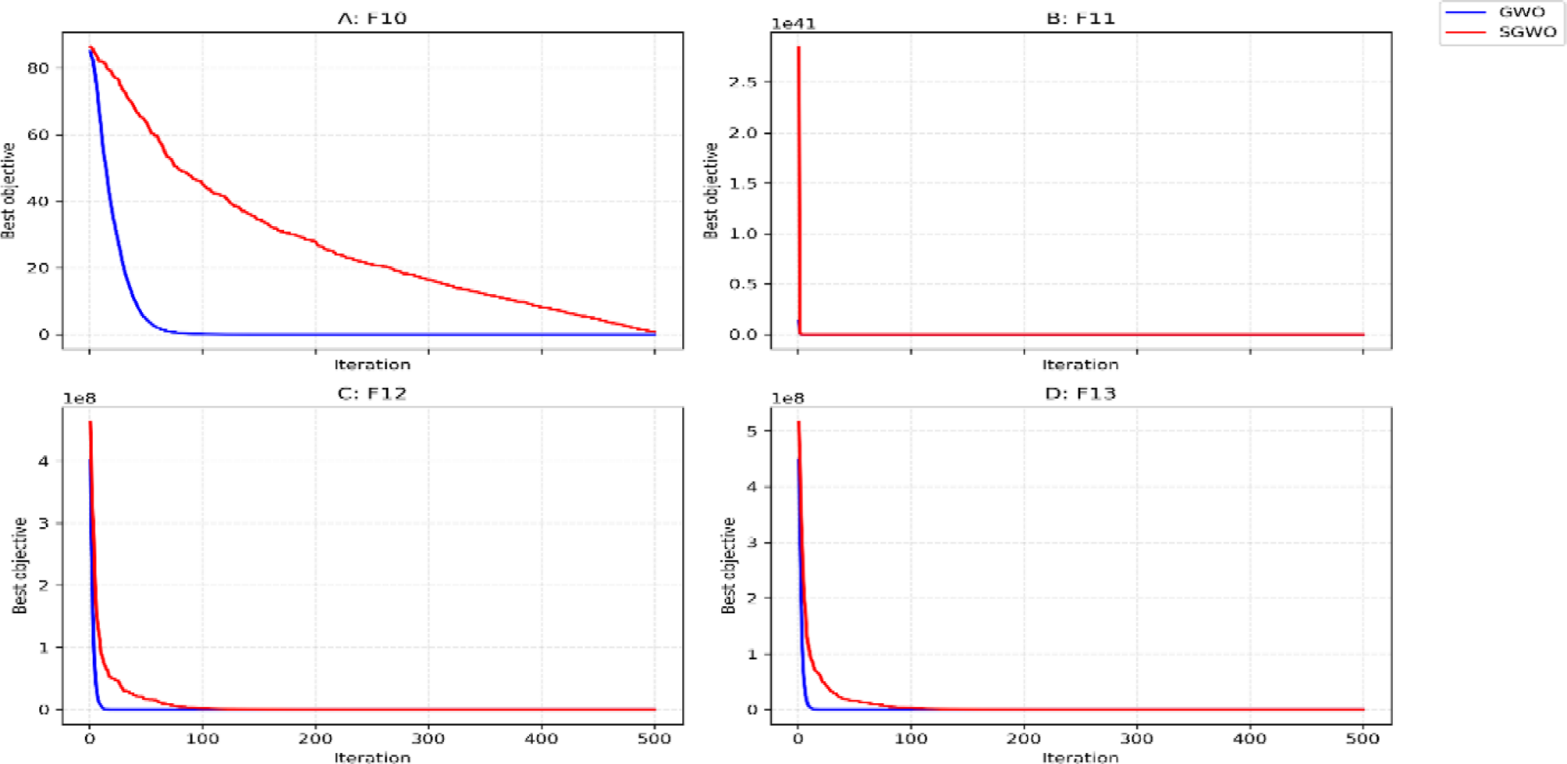
Convergence curves on F10–F13.

GWO achieves lower final errors on F10 and F11, confirming its strong exploitation on these penalized functions. Conversely, SGWO attains clearly lower objective values on F12 and F13, demonstrating a superior capability to escape local minima on more rugged and irregular search spaces.

**Fig. 12:**
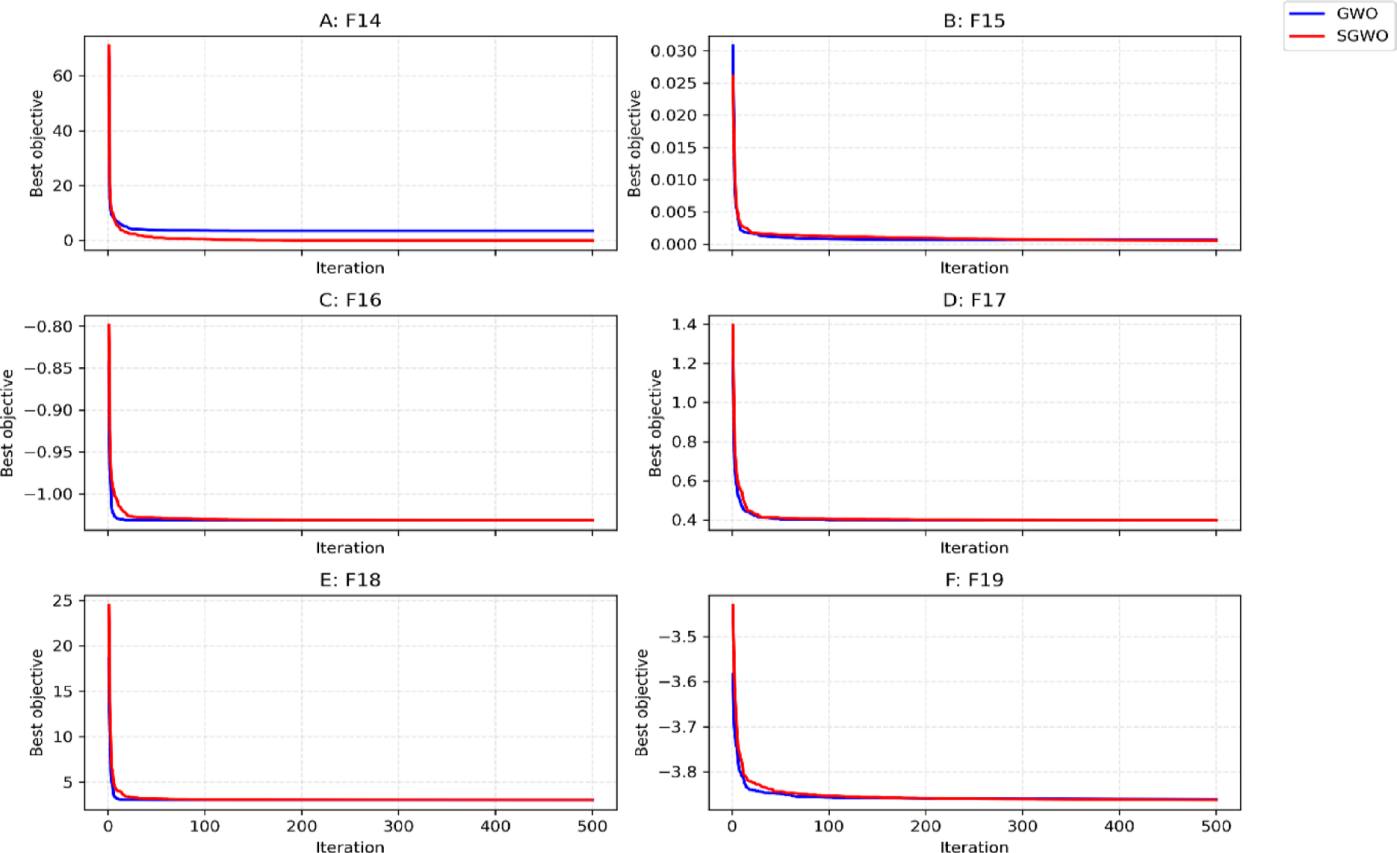
Convergence curves on F14–F19.

SGWO achieves lower final objective values on F14, F15, and F19, underlining its advantage on several fixed-dimension multimodal functions. GWO performs slightly better on F16 and F18, while both optimizers show almost identical behaviour on F17, where their final objective values are nearly indistinguishable.

#### Benchmark-function results

As shown in Table 12; Figs. 9, 10, 11, 12, both optimizers remained competitive across the selected benchmark functions, but their relative behavior depended on the landscape structure. In general, the canonical GWO showed stronger exploitation on smoother functions, whereas SGWO demonstrated a clearer advantage on several multimodal and more irregular functions. This pattern suggests that the stochastic perturbation improved search diversity and reduced premature concentration around suboptimal regions. These observations support the methodological motivation for transferring SGWO to the photovoltaic forecasting experiments.

### Main photovoltaic forecasting results

Table 13 reports the held-out test-set performance of all model–optimizer combinations on the photovoltaic forecasting task under the main experimental setting (EPOCHS = 5, iterations = 5, single run, SEED = 42). The reported metrics include MAE, MSE, RMSE, R², and Duration (s), providing complementary measures of forecasting accuracy and computational cost. MSE is reported as a squared-error metric and was computed from the corresponding RMSE values using MSE = RMSE². Under the same evaluation protocol, all compared configurations are reported, including the fixed manual baseline, Random Search, canonical GWO, and SGWO for both RNN and LSTM architectures.

**Table 13:** Test-set performance on the solar PV dataset under the main experimental setting (EPOCHS = 5, iterations = 5, single run, SEED = 42).

Model	HPO method	MAE	MSE	RMSE	R^2^	Duration (s)	Actual epochs
RNN	Baseline	0.053	0.006400	0.08	0.913	16.403	5
RNN	Random Search (Keras Tuner)	0.041	0.003721	0.061	0.95	147.531	5
RNN	GWO	0.038	0.006400	0.08	0.912	18.483	5
RNN	SGWO	0.037	0.004900	0.07	0.932	1411.194	5
LSTM	Baseline	0.025	0.002809	0.053	0.962	33.741	5
LSTM	Random Search (Keras Tuner)	0.021	0.003721	0.061	0.949	244.729	5
LSTM	GWO	0.019	0.001936	0.044	0.973	43.178	5
LSTM	SGWO	0.018	0.001681	0.041	0.978	2515.91	5

Table 13 shows that all evaluated configurations achieved strong predictive accuracy, with test R² values exceeding 0.91 in all cases. Nevertheless, the LSTM-based models consistently outperformed their RNN counterparts, indicating that the gated recurrent architecture is better suited to the nonlinear and temporally dependent structure of the PV forecasting task. Among all compared configurations, the SGWO-tuned LSTM achieved the best overall performance, with MAE = 0.018, MSE = 0.001681, RMSE = 0.041, and R² = 0.978. Relative to the manually tuned LSTM baseline, this corresponds to a 28.0% reduction in test MAE and a 22.64% reduction in test RMSE, together with an increase of 0.016 in R².

The comparison within the RNN group is more nuanced. SGWO achieved the lowest MAE among the RNN configurations, whereas Random Search (Keras Tuner) achieved the lowest RMSE and the highest R². This pattern indicates that hyperparameter optimization also benefits the simpler recurrent architecture, but the gains are less consistent than those observed for LSTM. Accordingly, the strongest conclusion supported by the main test-set results is not that SGWO dominates every configuration on every metric, but that the best overall configuration under the primary experimental budget is obtained by combining SGWO with LSTM.

**Fig. 13:**
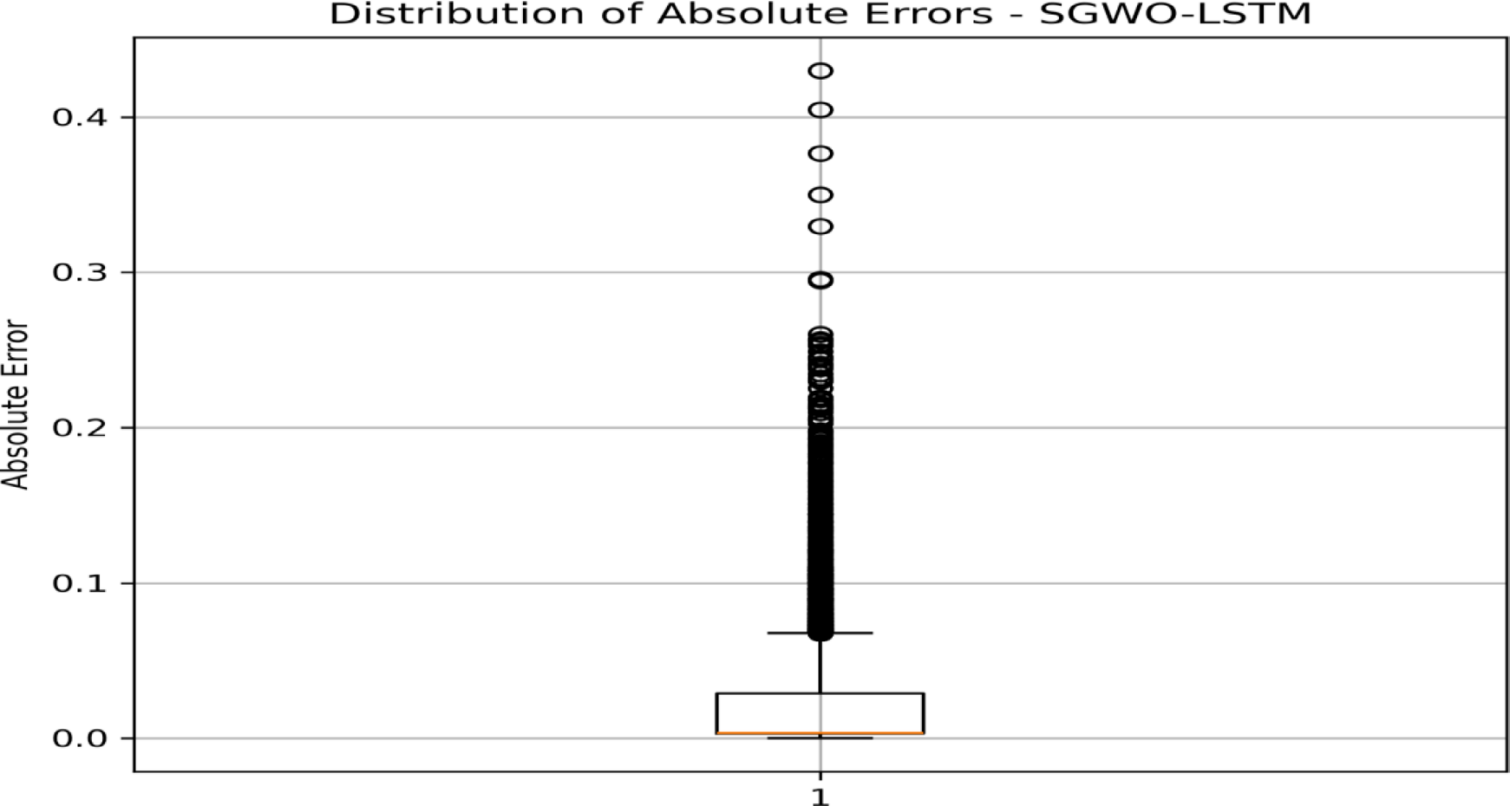
Boxplot of absolute errors for the SGWO-LSTM model on the held-out photovoltaic test set, summarizing the central tendency, dispersion, and outlier behavior of the prediction errors.

**Fig. 14:**
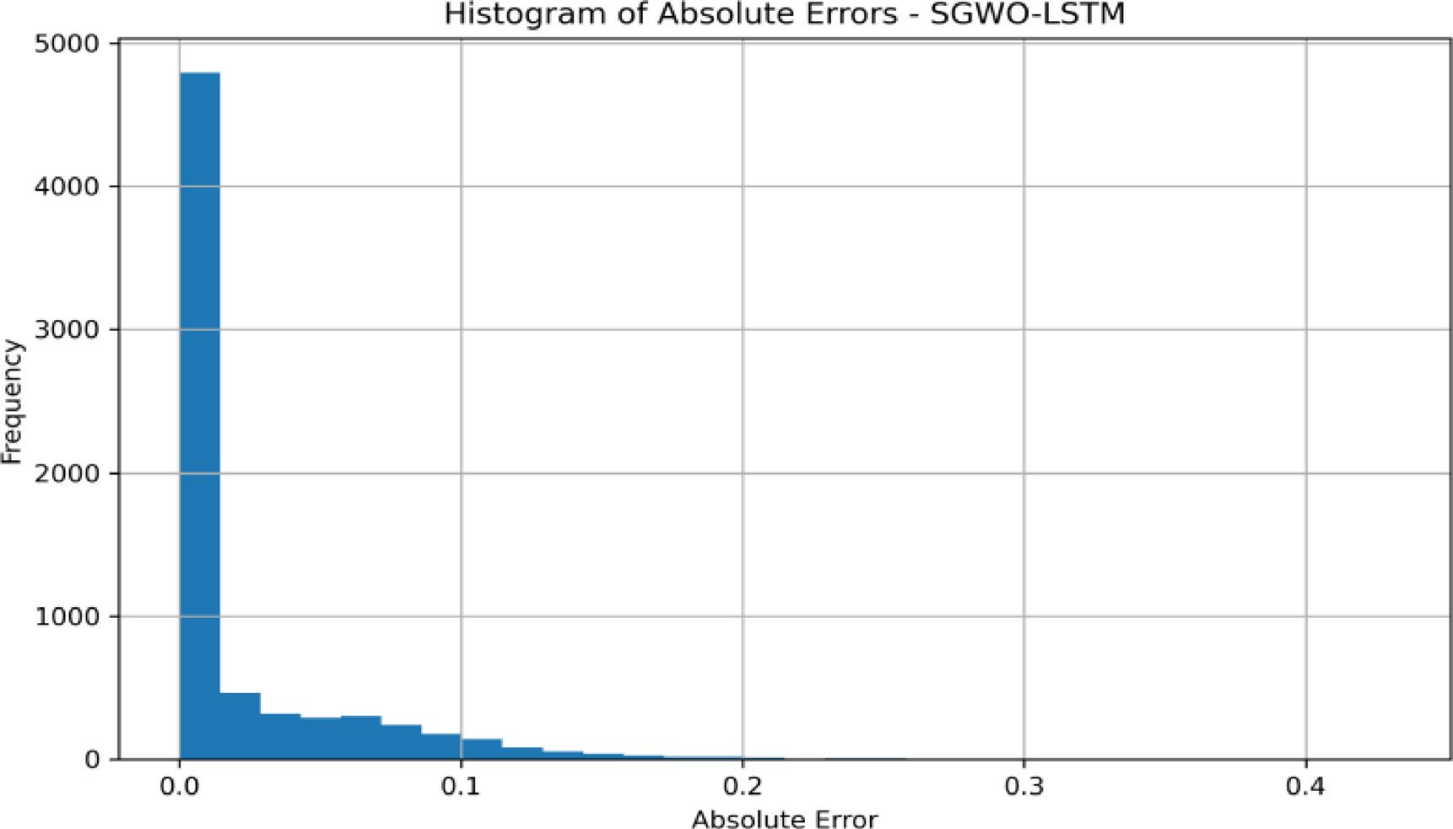
Histogram of absolute errors for the SGWO-LSTM model on the held-out photovoltaic test set, illustrating the overall distribution of prediction errors.

**Fig. 15:**
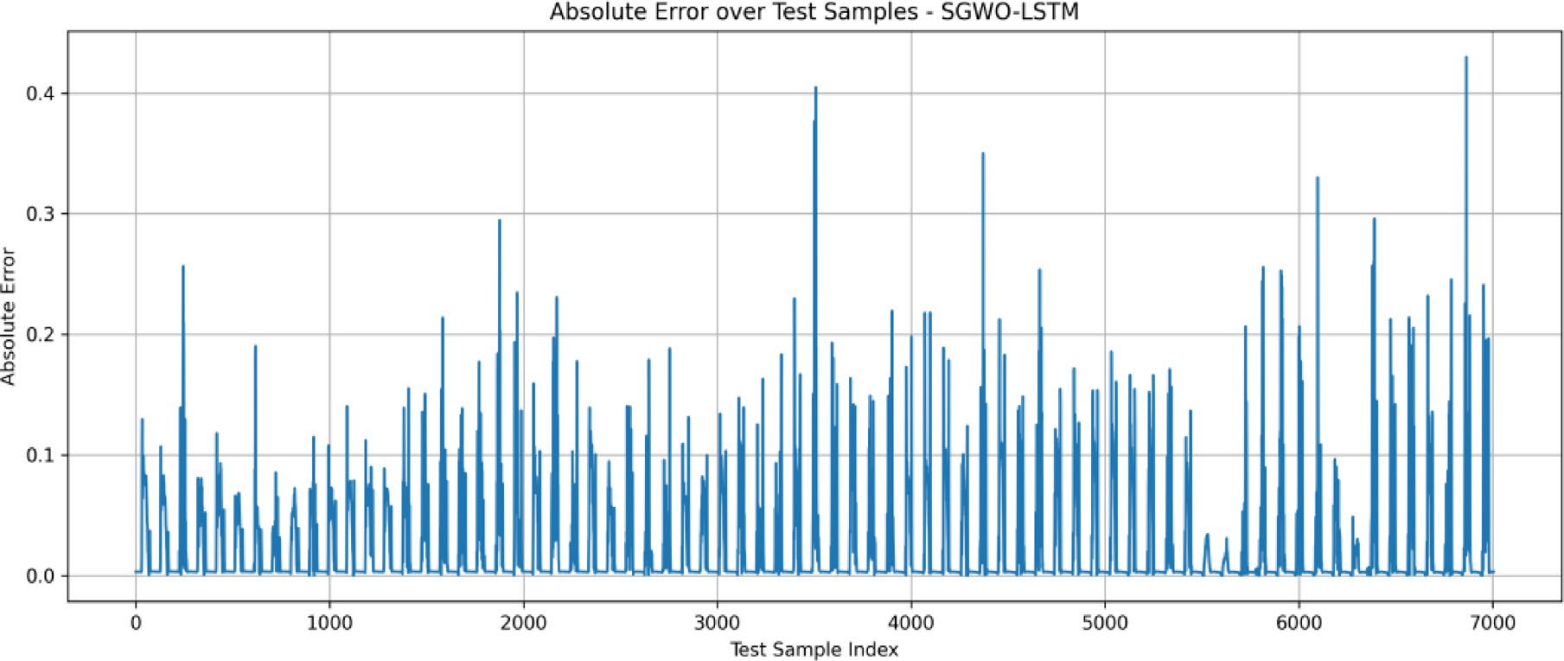
Absolute error across the held-out test samples for the SGWO-LSTM model, showing the temporal variation in prediction error magnitude.

**Fig. 16:**
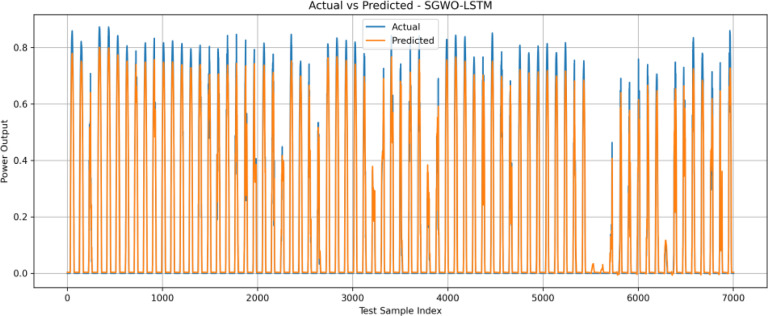
Actual versus predicted photovoltaic power output for the SGWO-LSTM model on the held-out test set.

The Duration column is retained to indicate the wall-clock computational burden of each method. As expected, SGWO requires longer runtime than the baseline and canonical GWO because it repeatedly evaluates candidate hyperparameter configurations through model training. These durations are implementation- and hardware-dependent and should therefore be interpreted together with algorithmic complexity analysis rather than as hardware-normalized efficiency measures. To complement the aggregate scalar metrics, absolute-error visualizations were generated for the held-out photovoltaic test set. The absolute error at time step *t* is defined as $\:\left|\:{y}_{t}-{\widehat{y}}_{t}\right|$, where $\:{y}_{t}$is the observed PV power output and $\:{\widehat{y}}_{t}$ is the corresponding SGWO-LSTM prediction. Figures 13, 14, 15 and 16 summarize the magnitude, distribution, temporal pattern, and prediction agreement of the SGWO-LSTM test errors.

### Sensitivity to the training budget

To evaluate the stability of the main conclusion, the experiments were repeated using larger maximum epoch budgets of 10, 50, 100, and 150 while keeping the intended hyperparameter-search budget fixed at five iterations. The corresponding test-set results are summarized in Appendix Tables A1, A2, A3, A4. The extended results indicate that the ranking of configurations is not invariant across training budgets. For the LSTM model, the best overall performance under the main experimental setting is obtained with SGWO at EPOCHS = 5. However, as the training budget increases, the baseline LSTM becomes comparable or superior, achieving MAE = 0.016, RMSE = 0.038, and $\:{R}^{2}=0.980$ at EPOCHS = 50, 100, and 150. For RNN, the strongest result also shifts across budgets: Random Search performs best at 5 and 10 epochs in terms of RMSE and R², the baseline is strongest at 50 epochs, and GWO performs best at 100 and 150 epochs. These results show that optimizer effectiveness depends on the interaction between model class, early stopping, and the available training budget. The most defensible conclusion is therefore a focused one: SGWO provides the best overall configuration under the main low-budget experimental setting and is particularly effective when coupled with LSTM at modest training budgets.

### Validation behavior and convergence analysis

This subsection uses validation RMSE and validation R² to assess relative training stability and generalization. Because the learning rate was selected during hyperparameter search and then kept fixed within each training run, the trajectories in these figures represent validation performance rather than an epoch-wise learning-rate schedule.

**Fig. 17:**
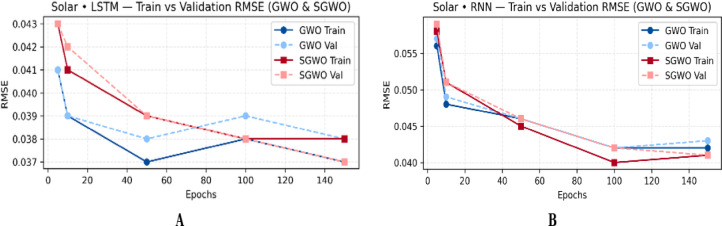
Training and validation RMSE trajectories for LSTM (**A**) and RNN (**B**) under the compared hyperparameter-optimization strategies.

Figure 17 compares the training and validation RMSE trajectories of the recurrent models under the evaluated optimization strategies. For the LSTM architecture, the SGWO-based configuration shows competitive validation behavior and relatively smooth error evolution in the main setting. For the RNN architecture, the behavior is more mixed, which is consistent with the more metric-dependent gains observed in the test-set comparison.

**Fig. 18:**
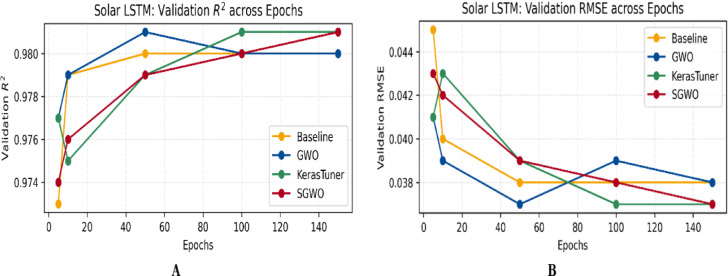
Validation performance of LSTM models across epoch budgets: validation R^2^ (**A**) and validation RMSE (**B**) for Baseline, Random Search (Keras Tuner), GWO, and SGWO.

**Fig. 19:**
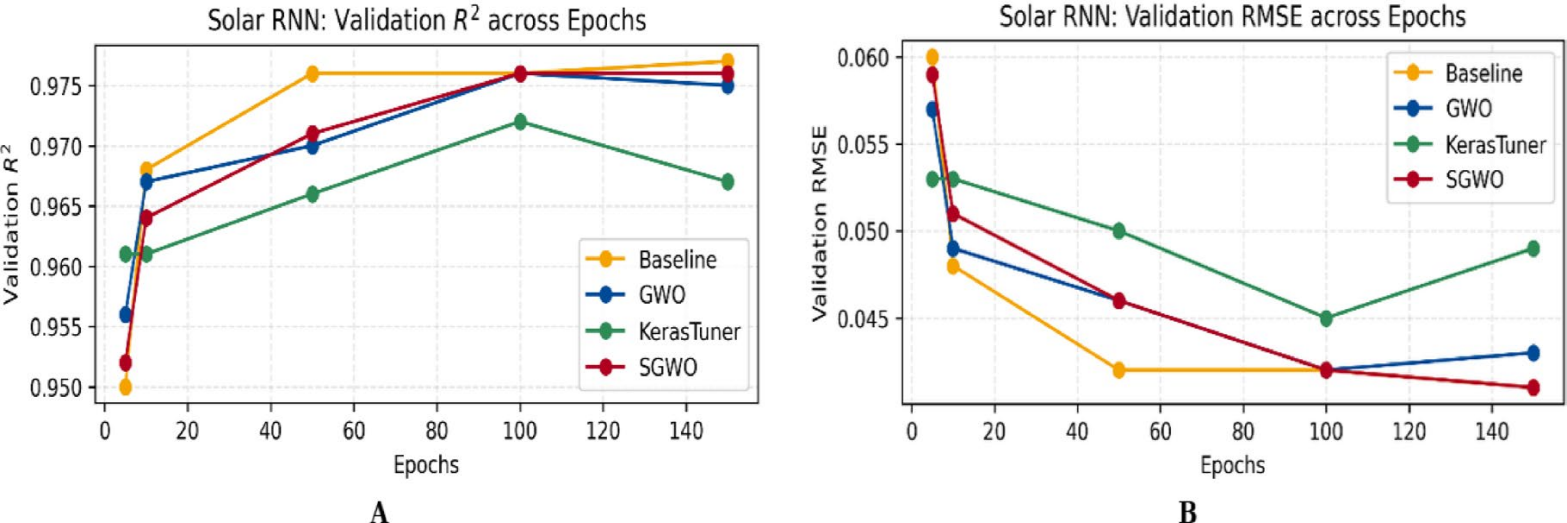
Validation performance of RNN models across epoch budgets: validation R^2^ (**A**) and validation RMSE (**B**) for Baseline, Random Search (Keras Tuner), GWO, and SGWO.

Figures 18 and 19 summarize the validation behavior of the compared optimization strategies across epoch budgets for LSTM and RNN, respectively. The results indicate that the relative ranking of the methods is not constant across all budgets. For LSTM, SGWO is highly competitive in the main low-budget setting, whereas the baseline becomes equally strong or stronger at larger epoch limits. For RNN, the strongest validation behavior also shifts across methods, indicating that optimizer effectiveness is conditional on the training regime rather than universally fixed.

### Challenges, limitations, and future research directions

Although the proposed SGWO-based hyperparameter-optimization framework achieved promising forecasting performance, several limitations should be acknowledged. First, the empirical analysis is based on a single PV dataset from a single data source and therefore constitutes a focused case study rather than a broad multi-site generalization analysis. Second, the experiments were conducted under a fixed chronological hold-out protocol rather than a rolling-origin or time-series cross-validation scheme. Third, the evaluation remains centered on point-forecast accuracy, while interpretability, uncertainty quantification, and feature-level sensitivity analysis remain limited. Fourth, hyperparameter optimization by metaheuristic search introduces substantial computational burden, especially as the search space, iteration budget, or model complexity increases. Finally, the present work does not include a direct techno-economic assessment .Future research should therefore investigate multi-site validation, broader temporal testing schemes, explainable forecasting analysis, probabilistic prediction, additional forecasting architectures such as transformer-based and graph-based models, and alternative hyperparameter-optimization frameworks.

## Conclusion

This study presented a stochastic Grey Wolf Optimizer (SGWO)-based framework for hyperparameter tuning of recurrent deep-learning models in photovoltaic (PV) power forecasting. The proposed approach was evaluated within a unified experimental setting that compared a fixed manual baseline, Random Search implemented through Keras Tuner, the canonical GWO, and the proposed SGWO for both RNN and LSTM architectures. The benchmark-function experiments showed that the relative behavior of the two wolf-based optimizers depends on the structure of the objective landscape: the canonical GWO generally showed stronger exploitation on smoother functions, whereas SGWO provided clearer advantages on several multimodal and more irregular functions, where the stochastic perturbation improved search diversity and reduced premature concentration around suboptimal regions. On the real-world PV forecasting task, the empirical results further demonstrated the practical value of this stochastic optimizer design. Among all evaluated configurations, the SGWO-LSTM model achieved the best held-out test performance, with MAE = 0.018, RMSE = 0.041, and R² = 0.978, while SGWO-RNN also improved upon the manual baseline and remained competitive with the other tuned alternatives.

Taken altogether, the outcomes of this research suggest that when a random (stochastic) perturbation is added to the GWO search procedure, it enhances the ability of recurrent deep learning-based forecasting models to generate accurate predictions while being robust within a consistent optimization protocol for hyperparameters. While the results of the present study are important, they need to be viewed from an appropriate perspective. The empirical validations were performed using only one actual world PV dataset under the same evaluation criteria. Therefore, no direct economic assessment of each model was made; no comparison was made regarding normalized computational efficiency; nor was a detailed feature level sensitivity study completed. Thus, further studies will provide a better understanding of how effective the proposed SGWO method is by evaluating and comparing its performance across multiple sites and different climate regions utilizing various cross-validation methods. In addition, as part of the comparison, multiple seed designs will be employed in order to increase the robustness of the evaluations. Finally, in addition to the comparisons already mentioned (i.e., cost awareness), a direct comparison will also be made to other types of forecasting models (e.g. transformers, graphs etc.) and different hyperparameter optimization techniques.

## Data Availability

The photovoltaic dataset analyzed in the current study is publicly available from the IEEE DataPort repository under the title “Renewable Energy Output Dataset of a Wind Turbine and Photovoltaic Station in Northern China” and is cited in the manuscript as Ref.^18^. In the present work, only the photovoltaic-related records and their associated meteorological variables were used.

## Appendix

### Appendix A. Supplementary tables for Section IV.C

The following compact tables summarize the held-out test-set metrics across the additional epoch budgets used for the sensitivity analysis. Each table retains the same metric set as Table 13 and reports the actual number of completed epochs when early stopping terminated training before the target epoch budget.

**Table A1:** Test-set performance at epoch = 10 (iterations = 5).

Model	HPO method	MAE	MSE	RMSE	R^2^	Duration (s)	Actual epochs
RNN	Baseline	0.037	0.004225	0.065	0.942	28.331	10
RNN	Random Search (Keras Tuner)	0.031	0.003025	0.055	0.959	222.067	10
RNN	GWO	0.03	0.003844	0.062	0.948	50.814	10
RNN	SGWO	0.036	0.005184	0.072	0.93	868.808	10
LSTM	Baseline	0.018	0.001849	0.043	0.975	46.073	10
LSTM	Random Search (Keras Tuner)	0.019	0.002116	0.046	0.971	371.813	10
LSTM	GWO	0.021	0.002025	0.045	0.972	37.444	10
LSTM	SGWO	0.02	0.002025	0.045	0.972	1378.813	10

Duration is reported in seconds. Actual epochs reflect the stopping point reached by the final selected configuration under early stopping.

**Table A2:** Test-set performance at epoch = 50 (iterations = 5).

Model	HPO method	MAE	MSE	RMSE	R^2^	Duration (s)	Actual epochs
RNN	Baseline	0.025	0.003025	0.055	0.959	115.413	44
RNN	Random Search (Keras Tuner)	0.033	0.005329	0.073	0.927	596.525	50
RNN	GWO	0.026	0.003249	0.057	0.956	35.91	25
RNN	SGWO	0.026	0.003136	0.056	0.957	8507.009	37
LSTM	Baseline	0.016	0.001444	0.038	0.98	137.295	39
LSTM	Random Search (Keras Tuner)	0.027	0.003249	0.057	0.956	1102.352	50
LSTM	GWO	0.016	0.001444	0.038	0.98	97.416	34
LSTM	SGWO	0.02	0.001936	0.044	0.973	13323.823	45

Duration is reported in seconds. Actual epochs reflect the stopping point reached by the final selected configuration under early stopping.

**Table A3:** Test-set performance at epoch = 100 (iterations = 5).

Model	HPO method	MAE	MSE	RMSE	R^2^	Duration (s)	Actual epochs
RNN	Baseline	0.025	0.003025	0.055	0.959	299.429	54
RNN	Random Search (Keras Tuner)	0.033	0.003844	0.062	0.947	5438.703	100
RNN	GWO	0.022	0.002401	0.049	0.967	1476.014	77
RNN	SGWO	0.029	0.003844	0.062	0.947	4458.123	100
LSTM	Baseline	0.016	0.001444	0.038	0.98	1237.713	66
LSTM	Random Search (Keras Tuner)	0.024	0.002809	0.053	0.962	8021.97	100
LSTM	GWO	0.018	0.001764	0.042	0.975	462.617	45
LSTM	SGWO	0.023	0.002601	0.051	0.965	8131.657	78

Duration is reported in seconds. Actual epochs reflect the stopping point reached by the final selected configuration under early stopping.

**Table A4:** Test-set performance at epoch = 150 (iterations = 5).

Model	HPO method	MAE	MSE	RMSE	R^2^	Duration (s)	Actual epochs
RNN	Baseline	0.029	0.004225	0.065	0.943	1199.534	150
RNN	Random Search (Keras Tuner)	0.031	0.004761	0.069	0.935	10627.771	97
RNN	GWO	0.027	0.003249	0.057	0.955	1350.413	79
RNN	SGWO	0.028	0.003721	0.061	0.95	5533.609	83
LSTM	Baseline	0.016	0.001444	0.038	0.98	1240.804	76
LSTM	Random Search (Keras Tuner)	0.024	0.002809	0.053	0.962	11617.898	111
LSTM	GWO	0.02	0.002025	0.045	0.972	772.07	71
LSTM	SGWO	0.019	0.002025	0.045	0.972	9518.34	150

Duration is reported in seconds. Actual epochs reflect the stopping point reached by the final selected configuration under early stopping.

## Code availability

The code used for preprocessing, sequence generation, model training, and hyperparameter optimization is available from the corresponding author on reasonable request.
